# Effect of Systematic Control of Pd Thickness and Annealing Temperature on the Fabrication and Evolution of Palladium Nanostructures on Si (111) via the Solid State Dewetting

**DOI:** 10.1186/s11671-017-2138-1

**Published:** 2017-05-19

**Authors:** Sundar Kunwar, Puran Pandey, Mao Sui, Quanzhen Zhang, Ming-Yu Li, Jihoon Lee

**Affiliations:** 1 College of Electronics and Information, Kwangwoon University, Nowon-gu, Seoul 01897 South Korea; 2 Institute of Nanoscale Science and Engineering, University of Arkansas, Fayetteville, AR 72701 USA

## Abstract

Si-based optoelectronic devices embedded with metallic nanoparticles (NPs) have demonstrated the NP shape, size, spacing, and crystallinity dependent on light absorption and emission induced by the localized surface plasmon resonance. In this work, we demonstrate various sizes and configurations of palladium (Pd) nanostructures on Si (111) by the systematic thermal annealing with the variation of Pd thickness and annealing temperature. The evolution of Pd nanostructures are systematically controlled by the dewetting of thin film by means of the surface diffusion in conjunction with the surface and interface energy minimization and Volmer-Weber growth model. Depending on the control of deposition amount ranging between 0.5 and 100 nm at various annealing temperatures, four distinctive regimes of Pd nanostructures are demonstrated: (i) small pits and grain formation, (ii) nucleation and growth of NPs, (iii) lateral evolution of NPs, and (iv) merged nanostructures. In addition, by the control of annealing between 300 and 800 °C, the Pd nanostructures show the evolution of small pits and grains, isolated NPs, and finally, Pd NP-assisted nanohole formation along with the Si decomposition and Pd-Si inter-diffusion. The Raman analysis showed the discrepancies on phonon modes of Si (111) such that the decreased peak intensity with left shift after the fabrication of Pd nanostructures. Furthermore, the UV-VIS-NIR reflectance spectra revealed the existence of surface morphology dependent on absorption, scattering, and reflectance properties.

**Electronic supplementary material:**

The online version of this article (doi:10.1186/s11671-017-2138-1) contains supplementary material, which is available to authorized users.

## Background

The fabrication of metallic NPs on semiconductors have attracted considerable research interests for various optoelectronic devices. Metallic NPs can demonstrate increased light absorption and emission owing to the localized surface plasmon resonance (LSPR) [1–3], and their optical properties can efficiently be tuned by the control of shape, size, and density of NPs [4–6]. Silicon is one of the most abundant semiconductor materials widely utilized for photovoltaic solar cells and photodetectors [7–10], and the performance of Si-based optoelectronic devices can be significantly improved by the integration of metallic NPs [11–16]. For instance, self-assembled aluminum (Al) NPs fabricated on Si can enhance the efficiency of solar cells owing to the LSPR exhibited by Al NPs [11]. Similarly, Si-based LEDs can produce a superior light emission via the excitation of surface plasmon resonance by silver (Ag) NPs [12]. Furthermore, systematically controlled orientation of Ag NPs grown on Si (100) and Si (111) can enhance PL intensity [13], and 3-D morphology of gold (Au) NPs on Si (111) demonstrates an increased light emission as compared to that of the 2-D flat layers [14]. Likewise, palladium (Pd) NPs, though not yet widely investigated, also have interesting plasmonic properties and thus applied in photo-catalysts, hydrogen-related sensing, and adsorption applications [17–19]. Taking these into account, the adaptation of Pd nanostructures on Si can offer promising opportunities for the Si-based applications; however, the systematic study on the fabrication of Pd NPs on Si (111) and its morphological and optical characterization is still quite deficient. In this paper, we demonstrate the systematical evolution of Pd nanostructures on Si (111) by the control of Pd deposition amount and annealing temperature. Depending upon the various deposition amount, the evolution of Pd nanostructures from small grains, NPs, elongated NPs, and merged nanostructures are evolved based on the thermal diffusion, Volmer-Weber growth model, and surface and interface energy minimization mechanism. On the other hand, the annealing temperature variation shows the distinctive evolution of Pd nanostructures such as tiny pits and grains, randomly distributed isolated NPs, and finally, Pd NP-assisted nanohole formation on Si (111). The effect of surface morphology evolution of Pd nanostructures on optical properties and Si lattice vibration modes are revealed by the reflectance and Raman spectra measurement.

## Methods

### Si (111) Substrate Preparation and Nanostructure Fabrication

In this work, the evolution of various configurations, sizes, and densities of Pd nanostructures was investigated on Si (111) (Silicon Materials Inc., Belarus) by the systematic control of Pd deposition amount at various annealing temperatures and annealing duration. Initially, the Si (111) substrates were diced into 1 × 1 μm^2^ by a machine saw. Then, the Si (111) were mounted on a holder for the degassing process at 750 °C for 30 min under 1 × 10^−4^ Torr in a pulsed laser deposition (PLD) system in order to clean the surface. Additional file 1: Figure S1 shows the Raman spectra of bare Si (111) in which the transverse acoustical (TA) mode peaks are observed at around 299.14 and 616.63 cm^−1^ and the transverse optic (TO) peaks are at 519.41, 950.40, and 973.83 cm^−1^. The clean Si (111) substrates after the degassing are now ready for the Pd deposition. In an ion coater chamber, the Pd films were deposited on Si (111) by the sputtering at a growth rate of 0.05 nm/s at the 3 mA ionization current under the vacuum of 1 × 10^−1^ Torr. Various thicknesses of Pd films between 0.5 and 100 nm were deposited by the systematic control of deposition time (20 s ~ 1 nm). Additional file 1: Figures S2 and S3 shows the surface morphologies of various Pd depositions and the cross-sectional line profiles, RMS roughness (Rq), surface area ratio (SAR), and height distribution histograms which show the gradual increase in the height distribution with the thickness. After the deposition of Pd on Si (111), the samples were introduced to the PLD chamber for the nanostructure fabrication. The annealing temperature was systematically increased to acquire the target temperature at a ramping rate of 4 °C/s under 1 × 10^−4^ Torr. The series of samples for deposition amount variation was annealed at three distinctive temperatures (450, 575, or 700 °C) for the constant annealing duration of 450 s. For the investigation of annealing temperature on evolution of Pd nanostructures, the annealing temperature was systematically varied between 300 and 800 °C, whereas deposition amount and annealing duration were set for 5 nm and 450 s respectively. The overall annealing process was controlled by the computer-operated recipe program in order to maintain the consistency. The temperature was immediately quenched down to the room temperature after the completion of each growth.

### Characterization of Pd Nanostructures

The surface morphologies of Pd nanostructures on Si (111) were characterized using an atomic force microscope (AFM) from the Park Systems Corp. (XE-70, South Korea) under an atmospheric condition. In order to minimize the tip effect, the tips from a single batch were used, having a length of 17–21 μm and a radius curvature less than 10 nm, force constant 40 nm^−1^, and resonant frequency ~300 kHz. After the characterization, the AFM images were analyzed using the XEI software (Park Systems) in terms of Rq, SAR, Fourier filter transform (FFT) spectra, and cross-sectional line profiles. The elemental analyses of the corresponding samples were carried out by using an energy-dispersive X-ray spectroscope (EDS) system with the spectral mode (Thermo Fisher Noran System 7, USA). The Raman and reflectance spectra were measured by UNIRAM II system (UniNanoTech Co. Ltd, South Korea), Andor shamrock sr500 spectrograph, laser −532 nm (for Raman), and deuterium and halogen light (for reflectance). The optical characterization was performed in dark room in order to avoid the external light interference.

## Results and Discussion

**Fig. 1:**
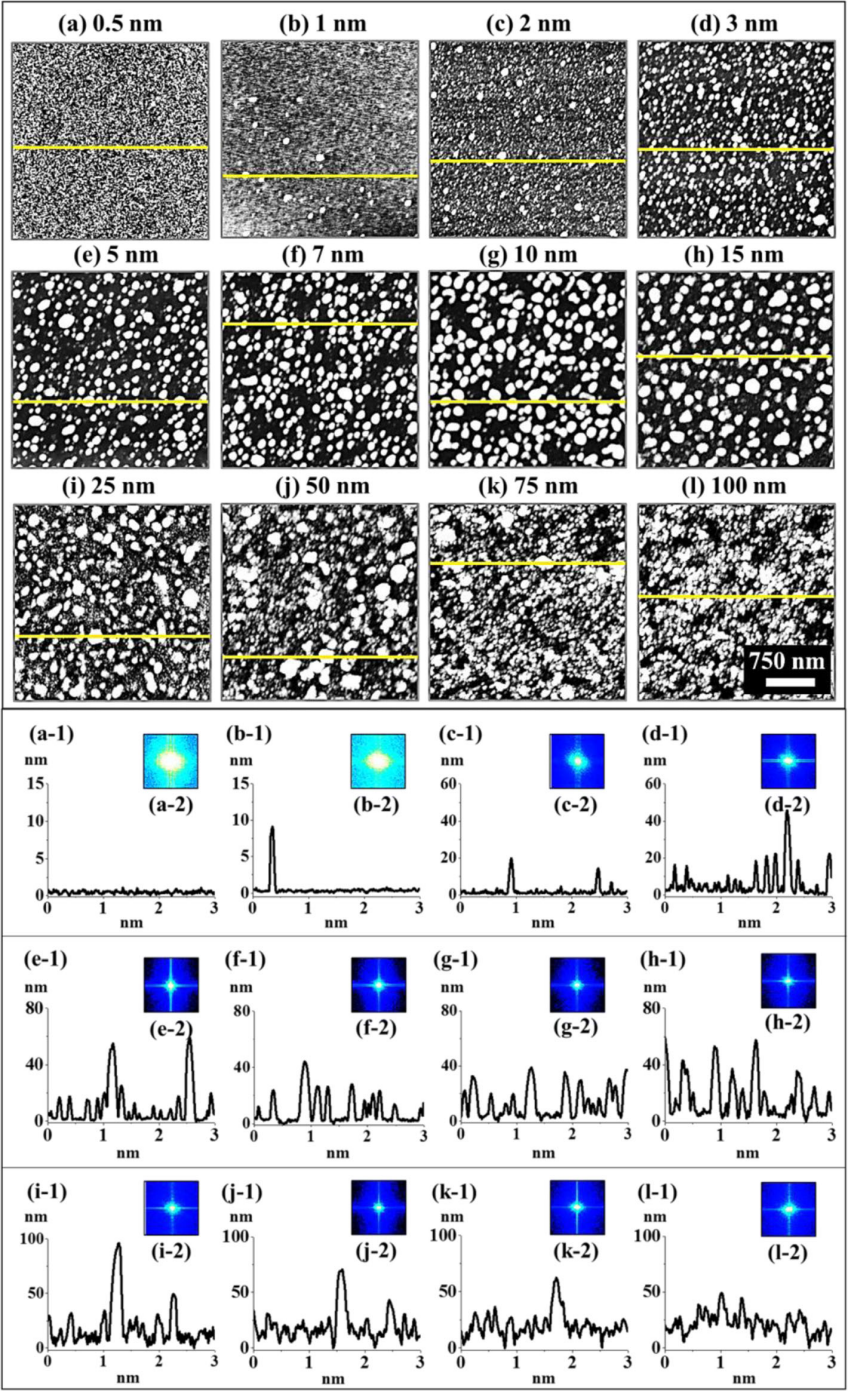
Pd nanostructures evolution on Si (111) by the variation of deposition amount between 0.5 and 100 nm via constant annealing at 575 °C for 450 s. AFM top-views of 3 × 3 μm^2^ shows the **a**–**c** nucleation of small Pd grains, **d**–**f** growth of Pd nanoparticles (NPs), **g**, **h** lateral evolution of Pd NPs, namely, formation of elongated Pd nanostructures, and **i**–**l** merged nanostructures. **a**-**1**–**l**-**1** Cross-sectional line profiles of AFM images. **a**-**2**–**l**-**2** Corresponding Fourier filter transform (FFT) spectra of AFM top-views

**Fig. 2:**
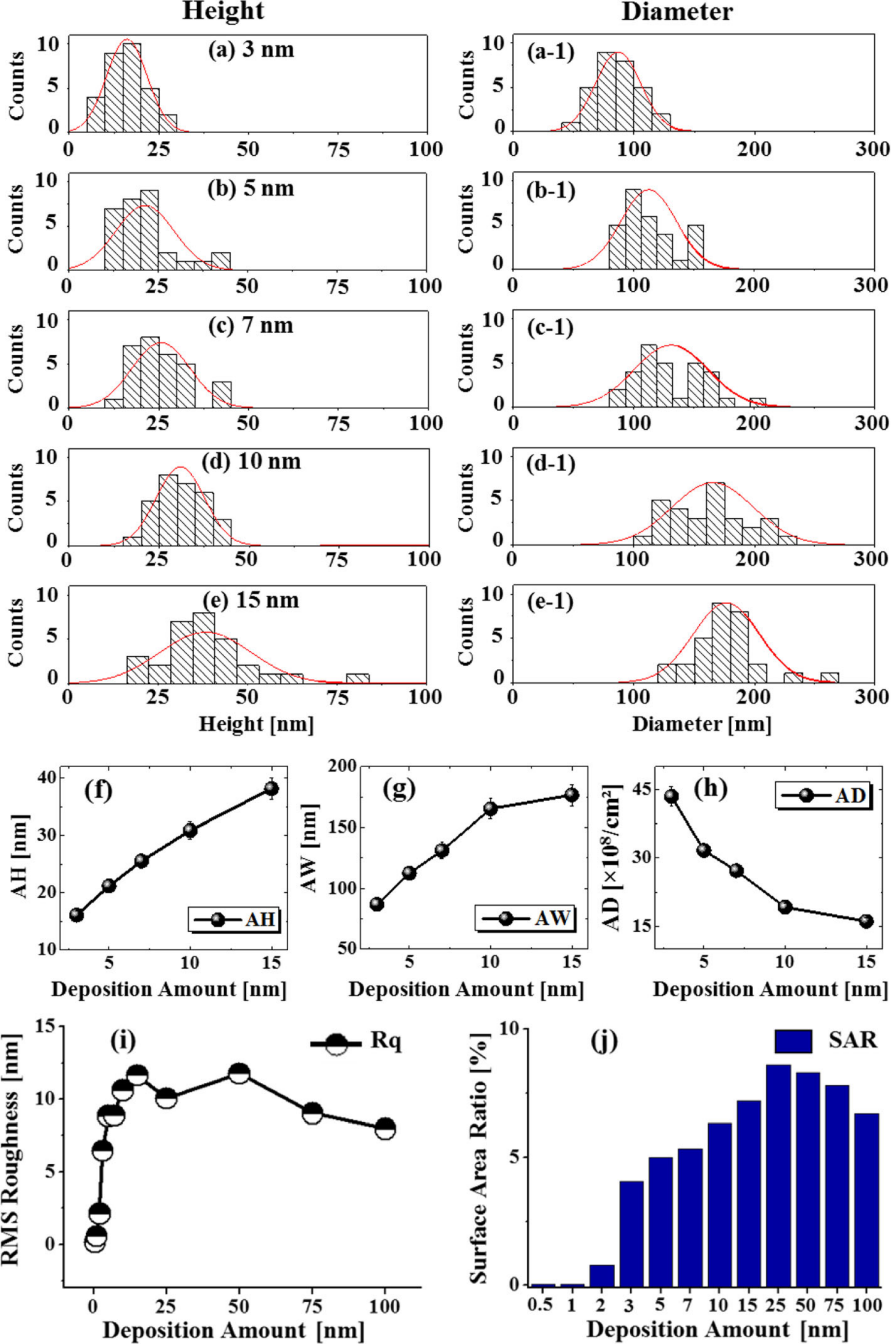
Height in **a**–**e** and diameter in **a-1**–**e-1** distribution histograms of Pd NPs with 3 to 15 nm deposition amount annealed at 575 °C for 450 s. **f**–**h** Summary of average height (*AH*), average width (*AW*), and average density (*AD*) with respect to the deposition amount. *Error bars* for the *AH*, *AW*, and *AD* are ±5% in **f**, **g**, and **h. i**, **j**. Summary plots of RMS roughness (*Rq*) and surface area ratio (*SAR*)

Figure 1 shows the formation of various sizes, configurations, and densities of Pd nanostructures on Si (111) with the control of Pd deposition amount between 0.5 and 100 nm annealed at 575 °C for 450 s. The detail of surface morphologies is presented along with the corresponding cross-sectional line profiles, FFT power spectra, and height and diameter histograms in Fig. 2. In general, four specific evolution regimes of Pd nanostructures were observed: 0.5–1 nm, formation of small pits and grains; 2–7 nm, nucleation and growth of Pd NPs; 10–15 nm, lateral evolution of Pd NPs, namely, formation of elongated nanostructures and 25–100 nm merged nanostructures. The overall evolution of Pd nanostructures can be explained based on the combinational effects of the thermal diffusion, agglomeration, Volmer-Weber growth, and surface and interface energy minimization mechanism. Initially, the surface morphology of Pd-deposited Si (111) showed a uniform distribution of Pd atoms all over the surface as shown in Additional file 1: Figure S2 and with the increased Pd deposition amount between 0.5 and 100 nm, their height distribution, Rq and SAR, were also gradually amplified as shown in Additional file 1: Figure S3. The thermal annealing of as-deposited sample induces the nucleation of Pd nanostructures due to the diffusion of Pd adatoms as activated by the sufficient thermal energy. The surface diffusion coefficient (*D*_s_) is temperature-dependent [20], and the diffusion length (*L*_D_) of adatoms is determined by the surface diffusion coefficient as *L*_D_ = √(*D*_s_ *t*), where *t* is the residence time [21, 22]. Thus, the diffusion length of adatoms can vary depending upon the annealing temperature. With a sufficient surface diffusion, metal adatoms can now nucleate and form nanostructures if being provided with the stronger binding between Pd adatoms as compared to the substrate atoms based on the Volmer-Weber growth model [23]. Once the nuclei are formed, they tend to grow larger by absorbing diffusing adatoms in order to reduce the surface and interface energy of the system and thus equilibrating the overall energy of thermodynamic system. As a result, self-assembled Pd NPs can be resulted on Si (111).

**Fig. 3:**
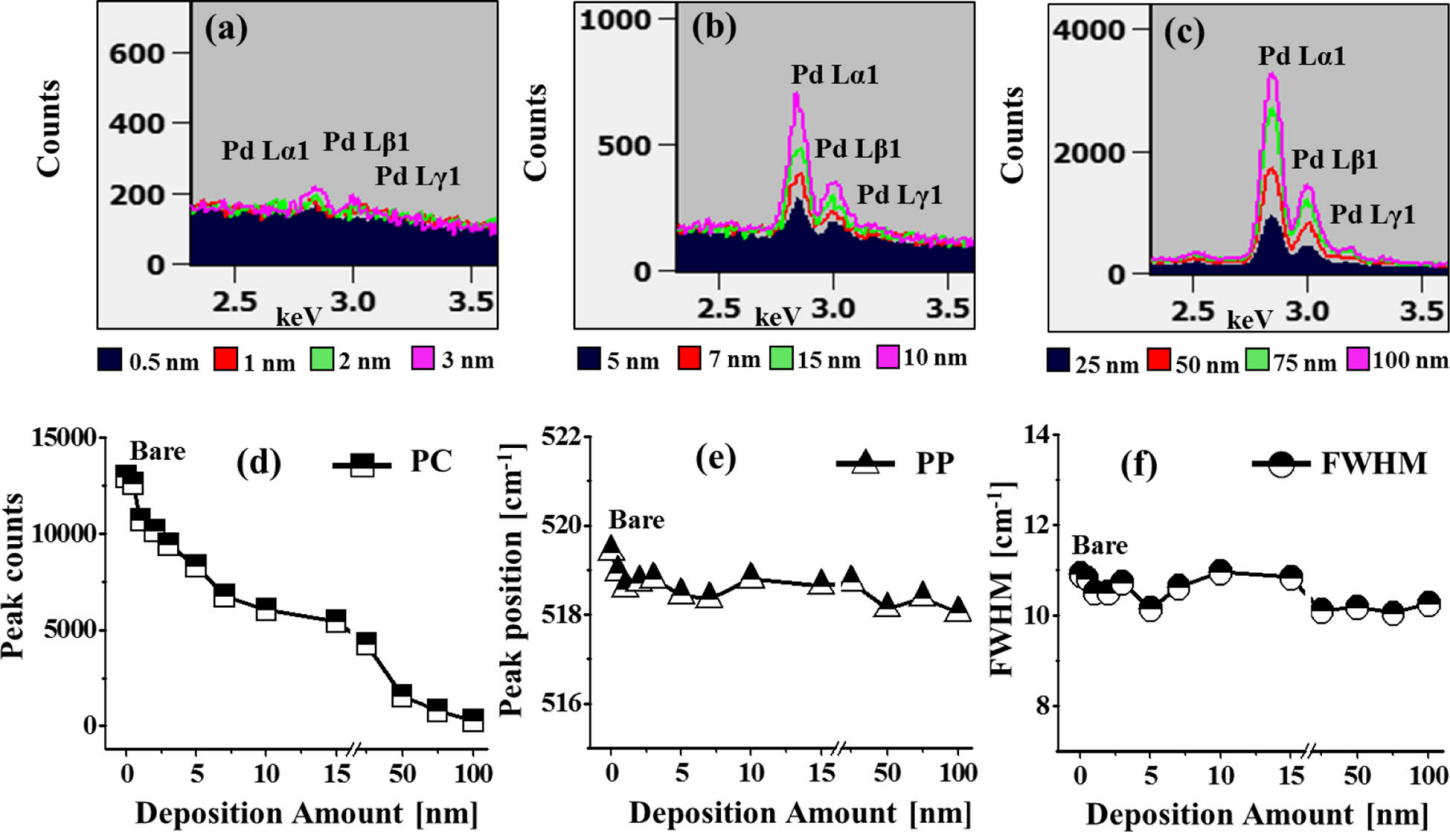
**a**–**c** Energy-dispersive X-ray spectroscopy (EDS) spectra of the samples with various Pd deposition amount between 0.5 and 100 nm after annealing at 575 °C for 450 s. Raman summary plots of **d** peak counts (*PC*), **e** peak position (*PP*), and **f** full width at half maximum (*FWHM*) as function of deposition amount between 0.5 and 100 nm. The full-range Raman spectra are shown in Additional file 1: Figure S6

In the meantime, the configuration, size, and density of nanostructures can be influenced by the systematic control of deposition amount between 0.5 and 100 nm at constant annealing environment. Initially, with the low Pd deposition amount 0.5 and 1 nm, the small pits and grains were formed as shown in Fig. 1a, b. Meanwhile, few relatively large grains were observed, which can be the consequence of different thermal expansion coefficient of Pd and Si (111). At 575 °C, thin Pd layer can diffuse and nucleate; however, due to insignificant amount of Pd adatoms, distinctive Pd NPs might not be formed. Thus, the surface became rougher and average height was slightly increased with the increased deposition amount from 0.5 to 1 nm as clearly demonstrated by the cross-sectional line profiles in Fig. 1a-1, b-1. The FFT power spectra also slightly shrunk in size due to the narrowed range of height distribution of the corresponding samples as displayed in Fig. 1a-2, b-2. The corresponding Rq and SAR were intensified from 0.15 to 0.55 nm and 0.029 to 0.047%, respectively, as shown by the summary plots in Fig. 2i, j and summarized in Additional file 1: Table S1. As the deposition was increased between 2 and 7 nm, the self-assembled isolated Pd NPs were fabricated as clearly shown in Fig. 1c–f and the growth of Pd NPs was preferential in the vertical direction as depicted by the line profiles. With the addition of Pd deposition amount, the Pd NPs further grew larger by aggregating nearby small NPs and adatoms until they reach an equilibrium state [24]. As a compensation, the density of NPs was decreased accordingly. When the NPs completely agglomerate with other small NPs, they grew vertically maintaining its dome shape to minimize the surface free energy. The cross-sectional line profiles drawn in typical regions reveal the gradually increased height in Fig. 1c-1–f-1. The FFT power spectra also became smaller with the fabrication of Pd NPs having larger size indicating the reduced surface height periodicity. Furthermore, the size evolution of Pd NPs was investigated by height and diameter distribution histograms along with the Gaussian distribution curve as shown in Fig. 2. In which, the range of height and diameter gradually increased and the peak of Gaussian distribution curve shifted to the right, denoting the enhanced dimension in lateral and vertical directions along with the deposition amount. In addition, the plots of average height, diameter, and density are presented in Fig. 2f–h, which show the inverse relationship between density and size evolution. At the same time, the Rq and SAR increased from 2.13 to 8.89 nm and 0.772 to 5.326% respectively. The Pd NPs kept on growing larger by minimizing its surface energy with increased Pd thickness. When the deposition amount was increased to 10 and 15 nm, lateral evolution of Pd NPs was observed, namely, elongated nanostructures. At the same time, the rate of dewetting can be slower and the attracting force between large NPs can be increased leading to the coalescence of NPs [24–27]. As a result, few elongated NPs were formed by the partial merging of the nearby NPs in order to reach low energy configurations. The height of Pd NPs was slightly increased for 10 nm as compared with 7 nm, and the lateral diameter was enlarged obviously with reduced density as shown by the AFM images and cross-sectional line profiles. Similarly, the FFT power spectra further got smaller. The average height and diameter histograms and plots also clearly depict the dimensional enhancement with deposition amount. The values of Rq and SAR also evidenced the size expansion of Pd NPs such that they were increased from 8.89 to 10.62 nm and 5.326 to 6.317% respectively. After the addition of Pd deposition amount between 25 and 100 nm, the coalescence or merging of NPs was further enhanced, resulting in the elongated Pd NPs and finally a layer-like structure as shown in Fig. 1i–l. As the dewetting rate can be significantly reduced with the increased thickness of Pd layer, the isolated Pd NPs may not be formed between 75 and 100 nm. The cross-sectional line profiles and FFT power spectra also illustrate the merged structures and the height reduction accordingly with increased deposition as clearly shown in Fig. 1i–l, i-1, l-1 respectively. The Rq slightly decreased to 10.05 nm with the deposition amount of 25 nm due to the height reduction of merged nanostructures, and Rq increased to 11.78 nm with the deposition amount of 50 nm which can be caused by the few large and isolated Pd NPs. Meanwhile, further increase in deposition amount to 75 and 100 nm resulted in the reduction of Rq to 9.04 and 7.98 nm due to the formation of layered structures. The SAR of the 25 nm sample was increased to 8.863% as compared with that of the preceding sample because of the merged nanostructures and small voids. As the deposition amount was increased to 50, 75, and 100 nm, the SAR was decreased to 8.289, 7.801, and 6.707%, respectively, suggesting the lateral growth and height reduction by the formation of merged nanostructures. Figure 3a–c shows the EDS spectra of corresponding Pd nanostructures on Si (111) between 2.3 and 3.6 keV. The EDS spectra show the gradually enlarged intensity of the Pd Lα1, Pd Lβ1, and Pd Lγ1 peaks accordingly with the increased Pd deposition amount between 0.5 and 100 nm. For example, the peak count was quite low at ~200 counts up to 3 nm in Fig. 3a and increased to ~750 with 10 nm in Fig. 3b and, finally, reached ~3500 with 100 nm in Fig. 3c, confirming the gradually increased deposition amount. Figure 3d, f shows the Raman spectra summary plots of peak counts (PC), peak position (PP), and full width at half maximum (FWHM) as function of deposition amount between 0.5 and 100 nm of the corresponding samples. It is clearly observed that the PC was gradually decreased and the PP was slightly left-shifted (~1 cm^−1^) while the FWHM was randomly varied over the range. The gradual decrease in PC can be attributed to the gradually increased surface coverage of Pd nanostructures with higher deposition amount, which can absorb incident photon as well as inhibit the interaction with substrate molecules. In terms of the PP, the bare Si (111) exhibits the five Raman bands at around 299.14, 519.41, 616.63, 950.40, and 973.83 cm^−1^ [28, 29] excited by the 532-nm laser as shown in Additional file 1: Figure S1 and the transverse acoustical (TA) mode Raman bands were observed at ~299 and 616 cm^−1^ and the transverse optic (TO) Raman bands were at ~519, 950, and 973 cm^−1^. The most intense TO Raman band at ~519 cm^−1^ was traced and summarized to characterize the samples, and the full-range spectra are shown in Additional file 1: Figure S6. From the bare, all of the samples showed a shift by ~1 cm^−1^ at ~518 cm^−1^ and the shift of such can be due to the strain developed by the lattice mismatch between the Pd nanostructures and substrate [30, 31].

**Fig. 4:**
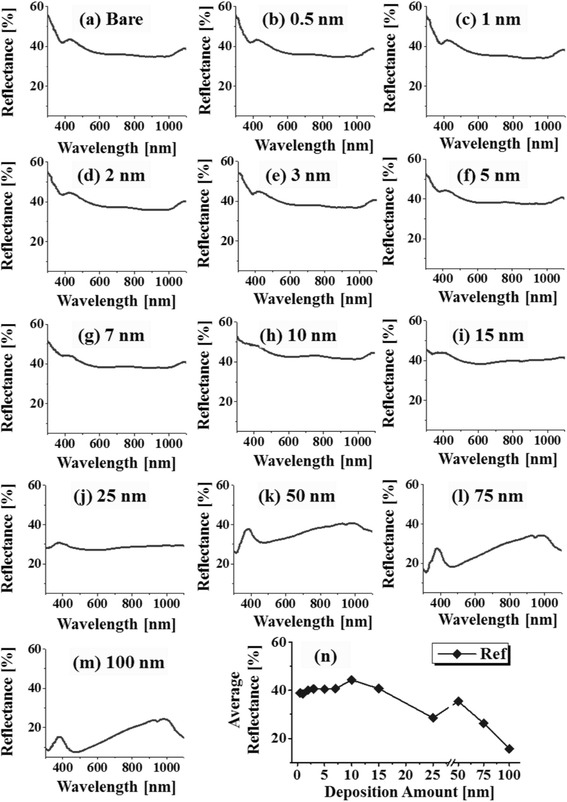
Reflectance spectra of the Pd NPs on Si with various deposition amount from 0.5 to 100 nm, annealed at 575 °C for 450 s. **a** Bare silicon. **b**–**m** Samples with Pd nanostructures with the deposition amount as depicted. **n** Summary plot of average reflectance with respect to the deposition amount

Figure 4 presents the reflectance spectra of Pd nanostructures on Si (111) as a function of wavelength ranging from 300 to 1100 nm. The bare Si (111) exhibits the average reflectance of ~38% (average value between 300 and 1100 nm) and is taken as a reference. In general, with the fabrication of Pd nanostructures, the average reflectance and spectral response was altered at different wavelength due to the effects such as absorption, reflection, and scattering enhancement of incident light based on the surface morphology of the Pd nanostructures [32, 33]. Meanwhile, there was no pronounced peak or valley formation in the specific wavelength region by the formation of the tiny Pd grains and large NPs as the spectral shape was identical with the bare Si as seen in Fig. 4b, i. However, the peak at ~427 nm was deteriorated with the higher thickness of Pd such as in between 7 and15 nm in Fig. 4g–i. As the Pd nanostructures and layer fully covered the surface, the reflectance of Si substrate might have been inhibited. In the case of Pd, the surface plasmon excitation in visible region is weaker and broader as compared to that of other metals such as Au and Ag [34–36]. Therefore, the resonance peaks are also weak and broader, in which, can make them indistinguishable for minor surface modification. Above 25 nm initial film thickness, the peak in the UV region (centered at ~380 nm), the wide valley in the visible region (centered at ~480 nm), and the broad shoulder in the NIR region were observed in Fig. 4j–m, which suggest the formation of absorption edge in the deep UV and visible wavelength. The formation of peak and absorption dip can be attributed to the surface plasmon resonance associated with the overgrown Pd NPs and voids [37]. The UV peak can be a quadrupolar resonance peak, whereas the NIR shoulder can be due to dipolar resonance, and both were found to be independent to the size and density of Pd NPs and voids. The specific average reflectance value is summarized in Additional file 1: Table S7. When the tiny pits and grain were evolved into the dense isolated Pd NPs, the average reflectance gradually enhanced as seen in Fig. 4n. Consequently, with the formation of round dome-shaped Pd NPs, the reflectance was increased which can be attributed to the high reflectivity of Pd nanostructures [38, 39]. However, the elongated and merged Pd nanostructures with wide spacing lowered the average reflectance above 15 nm, in which, the increased spacing between Pd NPs might have enhanced the light absorption by the coupling with surface plasmons. As the surface texture became rougher with the formation of voids and overgrown Pd layer between 15 and 100 nm, the incident photons can be absorbed or scattered resulting in the reduced average reflectance.

Meanwhile, for the identical deposition range, the temperature effect was investigated at the increased (750 °C) and decreased (450 °C) points, which are presented in the supplementary information in Additional file 1: Figures S7–S17. Overall, similar growth behavior was observed such that the size of the Pd nanostructures was increased, and density decreased accordingly with the increased deposition amount. As compared with the 575 °C set, the 700 °C set demonstrated slightly larger size of Pd nanostructures while the evolution trend was similar. The enhanced surface diffusion at 700 °C drove the Pd nanostructures to be a more stable configuration by minimizing surface energy such that the Pd NPs at 10 and 15 nm in the 700 °C set were much rounder. The evolution of surface morphology is also clearly evidenced by the cross-sectional line profiles, FFT power spectra, and the summary plots of Rq and SAR and summarized in Additional file 1: Table S4. The Raman spectra and their peak position, FWHM, and peak counts were also varied accordingly with the various morphologies of Pd nanostructures with increased deposition amount between 0.5 and 100 nm as clearly shown in Additional file 1: Figure S11 and summarized in Additional file 1: Table S5. The reflectance spectra in Additional file 1: Figure S12 also showed similar behavior in correlation with the surface morphology as described for the previous set. Meanwhile, annealing at comparatively low temperature of 450 °C resulted in the very small Pd grains on top over the Pd layer due to the limited diffusion of Pd adatoms as clearly illustrated by the AFM images, line profiles, FFT power spectra, RMS roughness, surface area ratio, EDS spectra, and Raman and reflectance spectra in Additional file 1: Figures S13–S17 and Additional file 1: Tables S4 and S5.

**Fig. 5:**
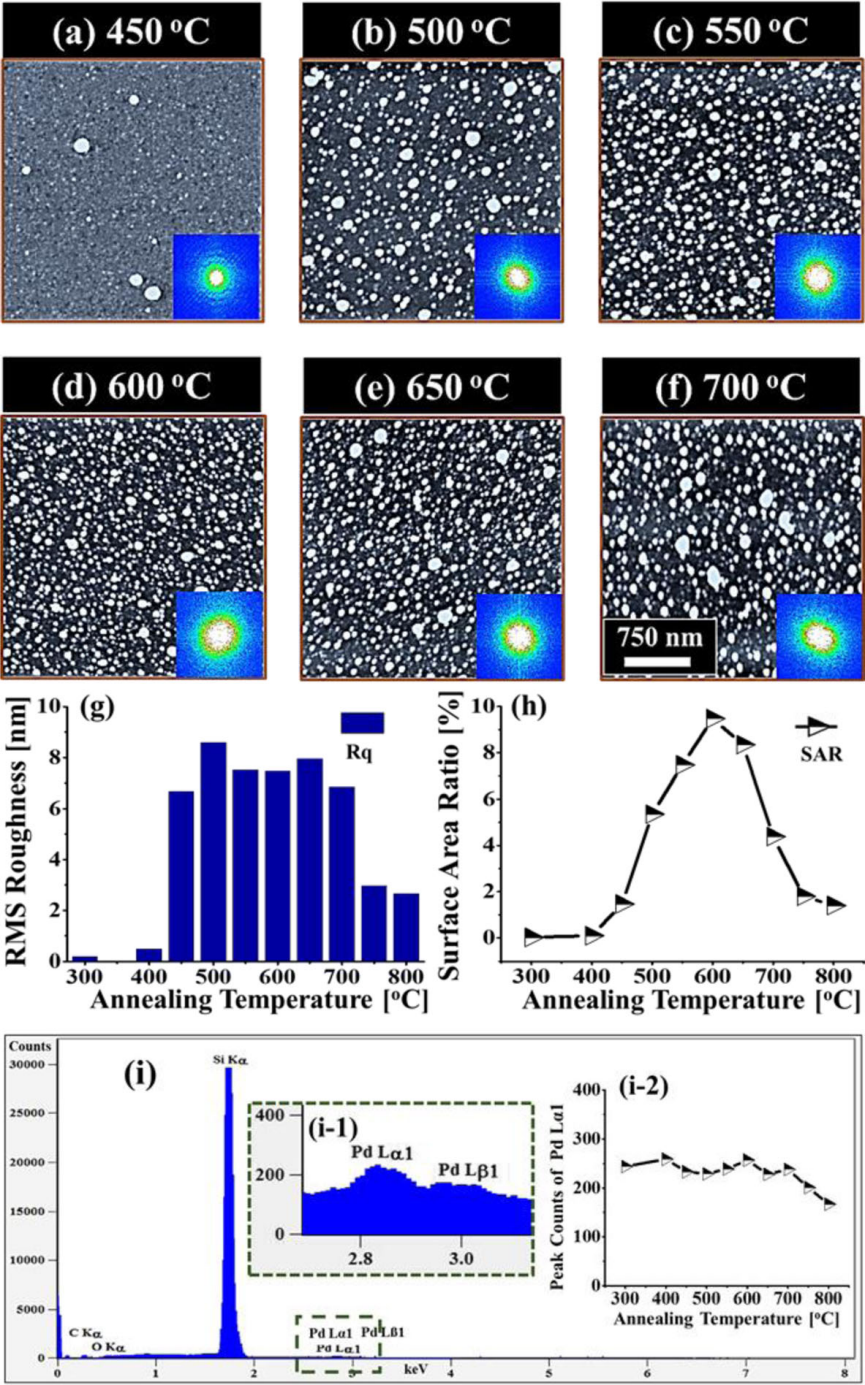
Effect of the annealing temperature (AT) between 450 and 700 °C on the evolution of self-assembled Pd NPs with constant deposition amount of 5 nm annealed for 450 s. **a**–**f** AFM top-views of Pd NPs with the area of 3 × 3 μm^2^, in which the *insets* represent the 2-D FFT power spectra. **g**–**h** Summary plots of Rq and SAR. **i** EDS elemental characterization. (*i-1*) Enlarged spectrum showing Pd Lα1 and Pd Lß1 peaks. (*i-2*) Summary plot of the intensity of Pd Lα1 with respect to the annealing temperature

**Fig. 6:**
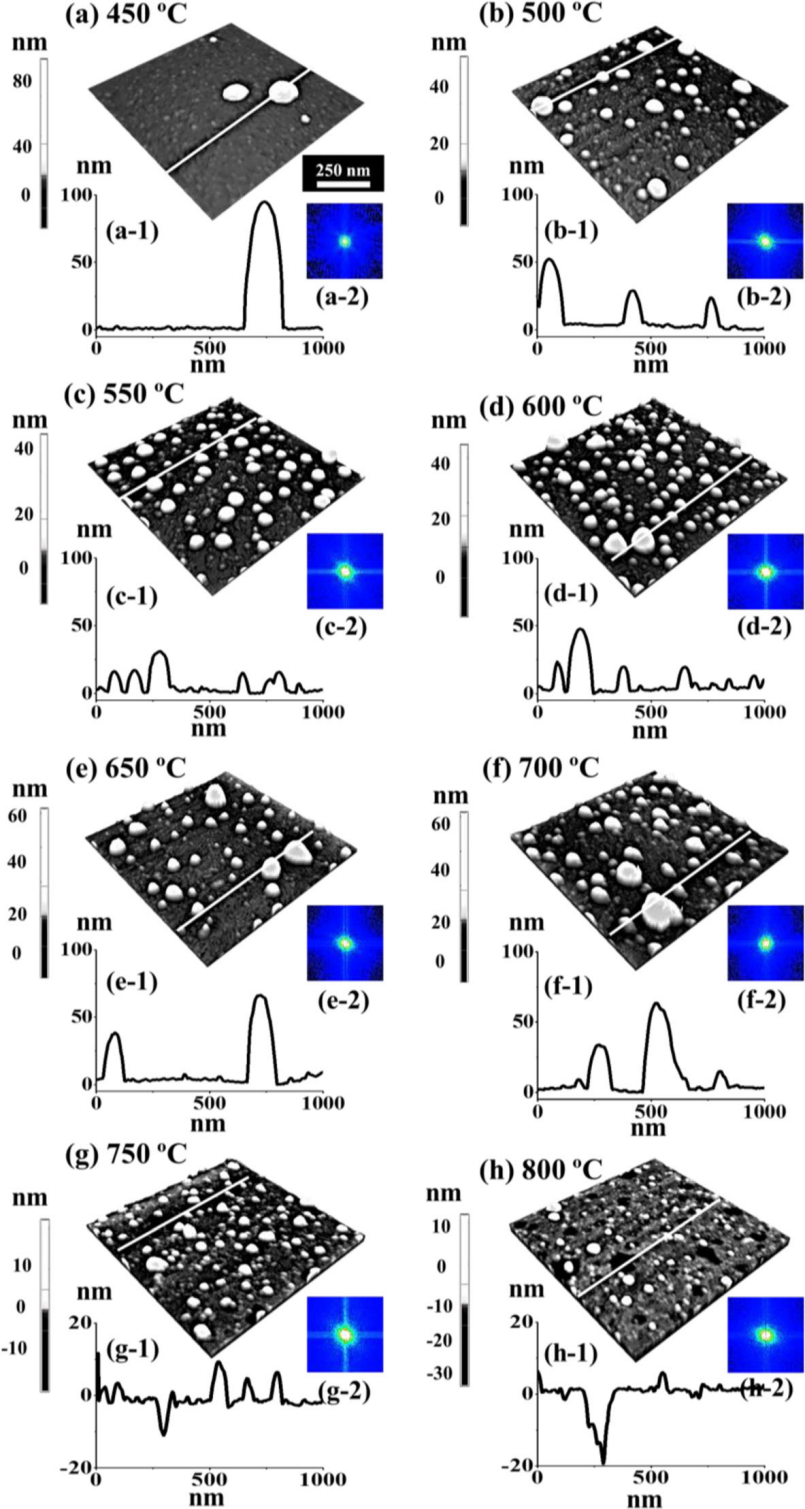
Evolution of the self-assembled Pd NPs and nanoholes on Si by varying the AT between 450 and 800 °C with 5 nm deposition amount. **a**–**h** AFM side-views of 1 × 1 μm^2^, with which the height of the Pd NPs is indicated with the *color bars*. (*a-1*)–(*h-1*) Cross-sectional line profiles acquired from the *white lines* in **a**–**h**. (*a-2*)–(*h-2*) Corresponding 2-D FFT power spectra

**Fig. 7:**
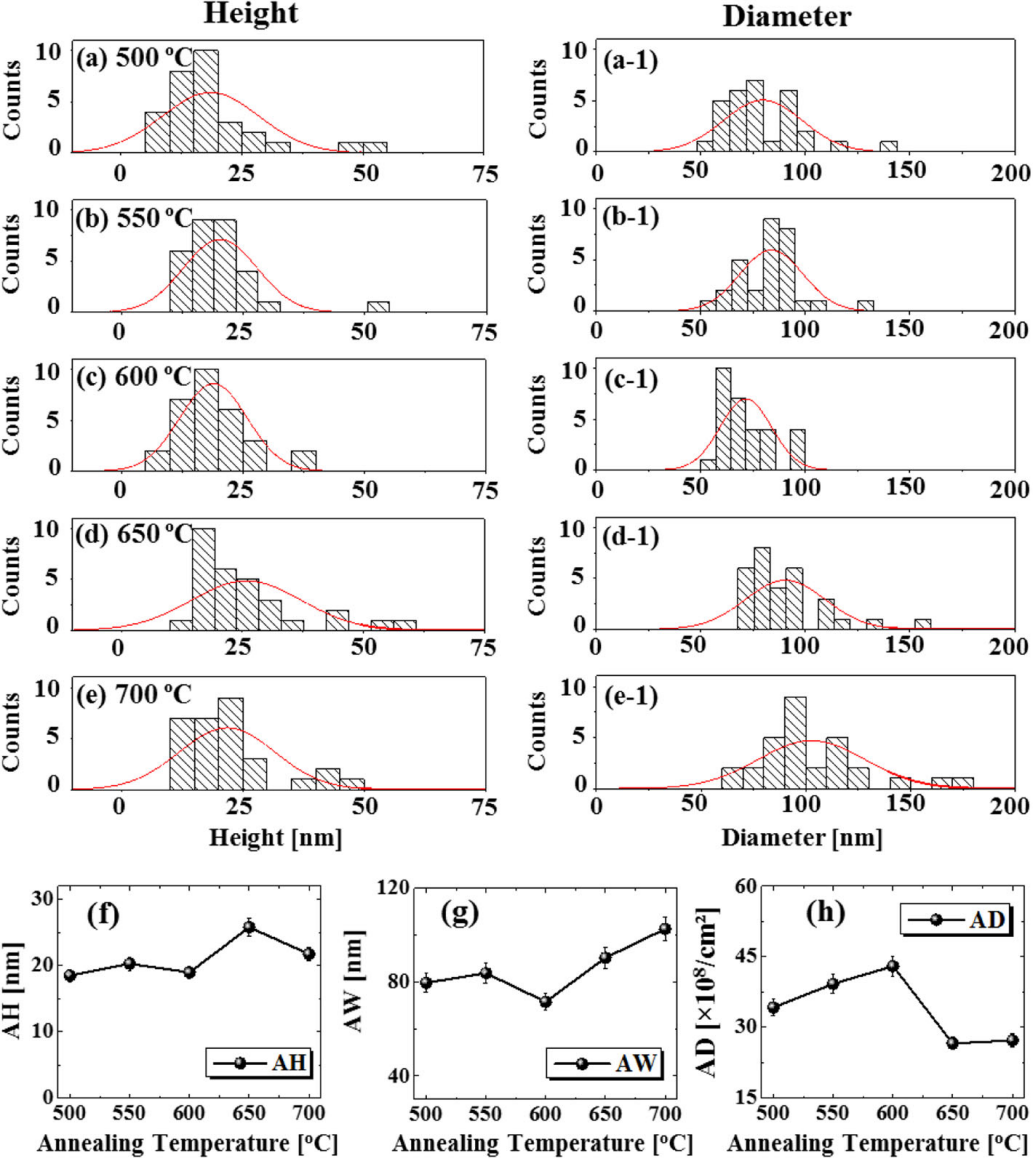
Height in **a**–**e** and diameter in **a-1**–**e-1** distribution histograms of the Pd NPs fabricated between 500 and 700 °C with 5 nm deposition amount. **f**–**h** Summary of *AH*, *AW*, and *AD* with respect to the deposition amount. *Error bars* for the *AH*, *AW*, and *AD* are ±5% in **f**, **g**, and **h**

Figures 5 and 6 show the evolution of self-assembled Pd NPs on Si (111) as a function of annealing temperature ranging from 450 to 800 °C with a fixed 5 nm Pd thickness annealed for 450 s. The morphological transformation of Pd NPs is analyzed by AFM top-views, side-views, cross-sectional line profiles, and Fourier filter transform (FFT) power spectra. The Pd NPs evolved into the various structural and spatial configurations (shape, size, and density) depending on the annealing temperature. More specifically, at lower thermal energy, the transition from the thin film to the Pd NPs occurred with the drastic evolution of surface morphology which evolved significantly, forming small isolated NPs. In contrast, at high thermal energy, an exceptional evolution process was observed, where the size and density of Pd NPs were sufficiently decreased and nanoholes were witnessed on the surface of Si. Furthermore, the density and size of nanoholes were enhanced with temperature, whereas the density and size of Pd NPs were decreased. As discussed earlier, the surface diffusion and nucleation of Pd NPs can be controlled by the thermal energy. The total surface and interface energy minimization of deposited thin film can lead to the enhanced agglomeration [40] or dewetting phenomena with the increasing temperature and decreasing film thickness. But in this case, the constant film thickness (5 nm) was maintained, and hence, the annealing temperature is responsible for the dewetting of Pd thin film. Firstly, the samples were investigated at lower annealing temperatures of 300 and 400 °C as shown in Additional file 1: Figure S18, which shows the early diffusion stage of thin film. The formation of tiny pits and grains at 300 °C were then grown gradually larger at 400 °C due to enhanced diffusion. As a result, the surface became rougher with the increased annealing temperature. Consequently, the tiny Pd NPs on the background with few large hillocks were observed at 450 °C and when the temperature was increased to 500 °C, randomly dispersed Pd NPs with various sizes were formed. The enhancement in the surface diffusion and agglomeration of Pd atoms resulted in the 3-D growth of NPs at an elevated temperature. The evolution of surface morphology is presented by the AFM side-views along with the cross-sectional line profiles and FFT spectra in Fig. 6. In cross-sectional line profiles, the average height was increased which depicts the size evolution of Pd NPs with temperature. The FFT power spectra also revealed the surface morphology as the size was gradually increased within the temperature range from 450 to 550 °C with increased height distribution of Pd NPs. Furthermore, the evolution of Pd NPs as shown by height and diameter distribution histograms along with Gaussian distribution curve in Fig. 7 displays the average size was increased between 500 and 600 °C. Similarly, the average density was also increased due to the enhanced agglomeration at higher temperature. The plots of Rq and SAR as function of annealing temperature in Fig. 5g, h demonstrated the evolution of surface condition along with the nanostructure formation. Up to 500 °C, the Rq was abruptly elevated due to the formation of small NPs and hillocks. The annealing between 300 and 600 °C caused the gradual increment in SAR as the total surface area was increased due to the formation of randomly distributed Pd NP density. When the annealing temperature increased from 600 to 700 °C, the size of Pd NPs became larger with reduced surface free energy, and at the same time, the density was correspondingly decreased. In case the temperature is below 600 °C, the dewetting process might not be completed; rather, Pd atoms were still agglomerating in order to form NPs. After annealing at 700 °C, the NPs were grown larger in size as evidenced by the average height of cross-sectional line profiles. The enhancement in the dimension can be proceeded to gain stable configuration with minimum surface energy. In addition, as shown by average height and diameter plot in Fig. 7f, g, at 600 °C, the average diameter and height were smaller and gradually increased up to 700 °C. Meanwhile, the density tends to decrease with temperature possibly due to the collision between diffusing Pd NPs. Annealing at high temperature between 750 and 800 °C leads to the formation of holes on the Si (111). The size and penetration depth of the holes were increased, whereas the height and density of the Pd NPs were decreased. The size reduction of Pd NPs at higher annealing temperature can be due to the penetration of NPs inside the Si (111) as a result of Si surface decomposition [41, 42]. Meanwhile, inter-diffusion of Pd and Si at the interface can occur resulting in the Pd-Si alloy system by lowering eutectic point [43, 44]. With increasing temperature, the Si surface decomposition and alloying of Pd-Si can be accelerated, which in turn drive the hole penetration. As shown in Fig. 6g, at 750 °C of annealing temperature, relatively small Pd NPs were dipped into the Si (111) substrate as observed in AFM side-views. With the annealing at 800 °C, the large Pd NPs also started to penetrate in to the Si (111) surface and large holes were formed. As observed in cross-sectional line profiles, the average height of Pd NPs was reduced, whereas the holes were deepened at higher temperature. Although the formation of holes and evaporation were progressive with annealing temperature, few large Pd NPs were still visible as large NPs were not completely dipped in Si as seen in Fig. 6h. The FFT power spectra showed the morphological change such that the surface height distribution was increased due to the digging of Pd NPs into the Si (111), and as a result, the round bright spot was slightly increased in size. Similarly, the SAR and Rq were simultaneously decreased with the surface modification due to the hole formation and reduced height of the Pd NPs. The SAR and Rq summary is presented in Additional file 1: Table S6. The elemental analysis of each sample was performed with EDS spectra that identifies the elements and amount present in the corresponding samples. As shown in Fig. 5i, i-1, two distinct peaks of Pd were observed, namely, Pd Lα1 and Pd Lβ1 at 2.845 keV and 3.028 keV respectively. The count plot of Pd Lα1 with respect to the annealing temperature is almost similar between the temperature range from 300 to 700 °C, which represents the equal amount of Pd in all samples regardless of different surface morphology after annealing. The counts of EDS spectra as shown in Fig. 5 (i-2) were reduced as compared to the samples at lower annealing temperature. This can be due to the penetration of Pd NPs inside Si, which reduce the interaction of Pd with incident X-ray resulting in the reduced EDS count. The full-range EDS spectra of the individual samples are presented in Additional file 1: Figure S19.

**Fig. 8:**
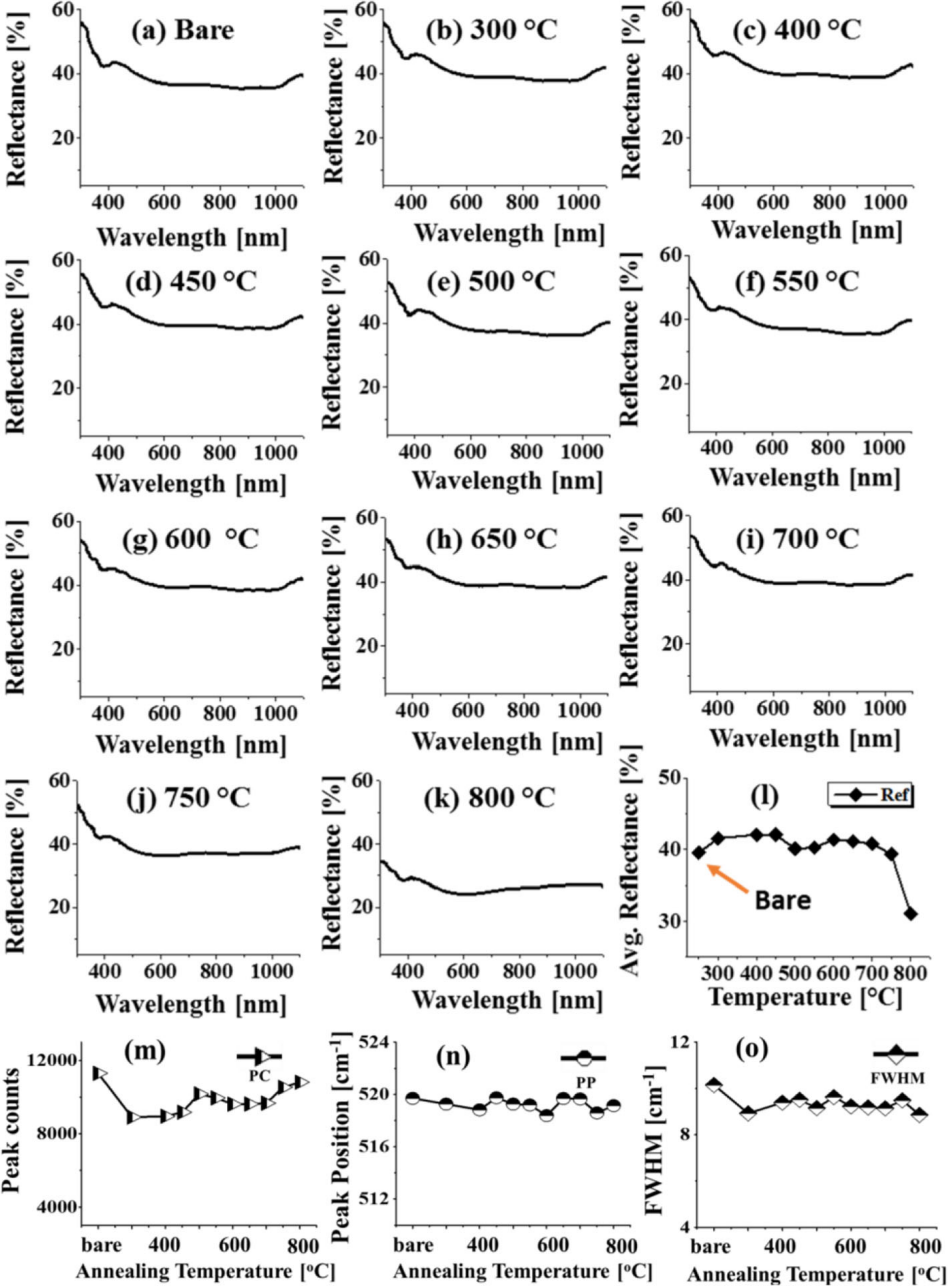
Reflectance spectra of the Pd nanostructures on Si (111) fabricated at varying temperatures. **a**–**k** Bare Si (111) and samples annealed at 300–800 °C for 450 s with 5 nm of Pd deposition amount. **l** Summary plot of average reflectance. **m**–**o** Summary plots of *PC*, *PP*, and *FWHM* of the Raman spectra as a function of the annealing temperature

Figure 8 shows the reflectance spectra within the wavelength range from 300 to 1100 nm. Generally, the average reflectance of Pd/Si (111) was slightly higher than that of bare Si (111) as shown in Fig. 8b–k. With an increase in annealing temperature from 300 to 450 °C, only pits, grains, and few NPs on surface were evolved, and consequently, the average reflectance also slightly increased as a result of enhanced reflection as discussed earlier [37]. Above 500 °C of annealing, the surface consisted of isolated Pd NPs and the total surface coverage was reduced as the tiny NPs on the background combined with large ones. As a result, the average reflectance can be slightly reduced at 500 and 550 °C. At 600 °C, the average reflectance was slightly increased and remained almost similar up to 700 °C because of the similar morphology of Pd NPs. However, as the hole formation on Si (111) has started at 750 °C, the average reflectance tends to decrease likely due to the light trapping effect which became more effective with the plenty of hole formation at 800 °C; as a result, the average reflectance was sharply decreased. The light trapping occurs due to the multiple light reflections inside the nanoholes that increases optical path of incident light [45]. In this case, the spectral shape remained almost constant indicating no significant enhancement by the interaction of surface plasmon and incident light. Furthermore, the Raman characterization of the samples was performed. As discussed, due to the lattice mismatch and the different thermal expansion coefficients between the substrate and Pd nanostructures, the Raman peaks or vibration modes (TO and TA) were altered as shown in Fig. 8m–o. The peak counts (intensity) were at maximum for bare Si (111) and dropped to minimum for the samples at 300 °C. Later, with the increment of the annealing temperature up to 800 °C, the intensity was gradual with the reduced surface coverage. At the same time, the peaks were slightly left-shifted except that these were bounced toward high frequency in some samples. The FWHM correspondingly left-shifted as represented by the plot in Fig. 8d. The overall shift appeared in the peak position, and full width at half maximum (FWHM) can be the consequence of lattice mismatch due to the formation of Pd nanostructures as discussed.

## Conclusions

In this work, the fabrication of Pd nanostructures with the various configurations, sizes, and densities was successfully demonstrated with the systematic control of deposition amount and annealing temperature on the morphology of Pd nanostructures on Si (111). Upon the annealing at temperatures (575 and 700 °C), the systematic increase in deposition amount resulted in the four distinctive configurations of Pd nanostructures such as small pit and grain formation, nucleation and growth of Pd NPs, lateral evolution of Pd NPs, and finally, merged nanostructures. The evolution of various configurations of Pd nanostructures were discussed based on the thermal diffusion, Volmer-Weber growth model, and surface and interface energy minimization mechanisms. Moreover, the incremental variation of annealing temperature demonstrated the formation of tiny pits and grains at relatively low temperature (300 to 400 °C), randomly distributed Pd NPs (450 to 700 °C), and finally, Pd NP-assisted hole formation at high temperature (750 to 800 °C). Depending on the surface conditions, the Raman spectra revealed the decreased intensity and left shift of phonon modes of Si (111) as correlated to the average surface coverage of Pd nanostructures and the stress between Pd and Si. Moreover, the reflectance spectra were dependent with the size and average surface coverage of Pd nanostructures that modulates the average reflectivity, absorption, and scattering. Finally, this study can provide the simple approach of fabricating Pd nanostructures with distinctive and controllable physical structures thereby exploiting related properties for the range of catalytic- and plasmonic-related application. In addition, the Pd NP-assisted porous Si formation can be achieved for antireflective Si substrates.

## Additional files

Additional file 1: Figures S1–S19. and Tables S1–S10. Supplementary materials include additional AFM images, EDS spectra, reflectance spectra, and Raman spectra of various Pd NPs. (DOCX 27737 kb)
