# Supplementary material for: Effect of Systematic Control of Pd Thickness and Annealing Temperature on the Fabrication and Evolution of Palladium Nanostructures on Si (111) via the Solid State Dewetting
Source: Nanoscale Res Lett. 2017 May 19;12:364. doi: 10.1186/s11671-017-2138-1 (PMC5438330; doi:10.1186/s11671-017-2138-1)
Supplement: Supplementary file 1 — Supplementary materials include additional AFM images, EDS spectra, reflectance spectra, and Raman spectra of various Pd NPs. (DOCX 27737 kb) [file 11671_2017_2138_MOESM1_ESM.docx]

**Additional file**

***Effect of Systematic Control of Pd Thickness and Annealing Temperature on the Fabrication and Evolution of Palladium Nanostructures on Si (111) via the Solid State Dewetting***

**Sundar Kunwar^1^, Puran Pandey^1^, Mao Sui^1^, Quanzhen Zhang^1^, Ming-Yu Li^1^ and Jihoon Lee^1,2*^**

^1^ College of Electronics and Information, Kwangwoon University, Nowon-gu Seoul 01897, South Korea

^2^ Institute of Nanoscale Science and Engineering, University of Arkansas, Fayetteville AR 72701, USA. Correspondence e-mail: jihoonleenano@gmail.com

**
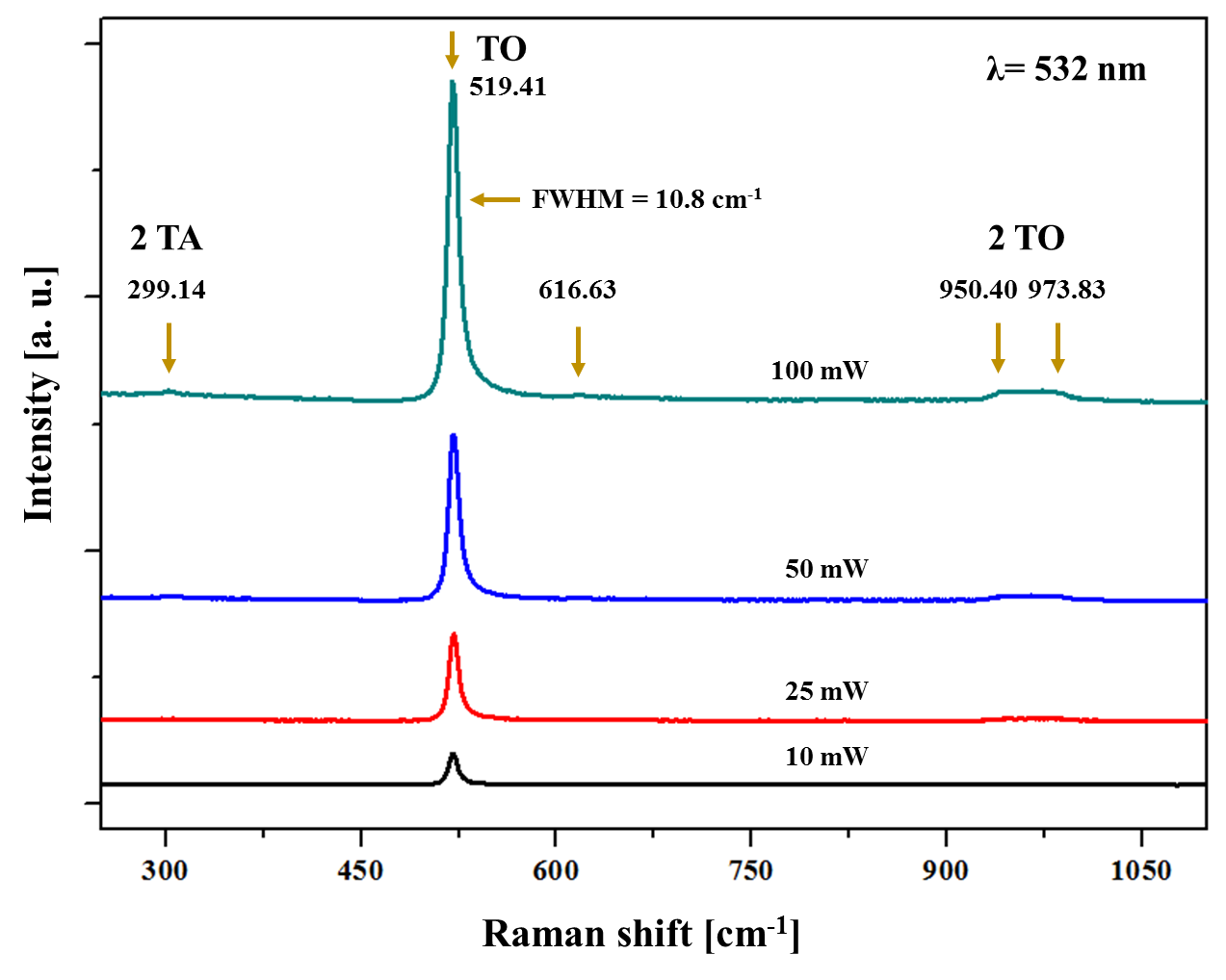
**

**Figure S1:** Raman spectra of bare Si (111) at room temperature excited by a continuous wave (CW) laser of 532 nm with the variant power of 10, 25, 50 and 100 mW. The Si peaks are denoted by arrows. The transverse acoustical (TA) mode peaks are observed at ~ 299.14 and 616.63 cm^-1^. The transverse optic (TO) peak is at ~ 519.41 cm^-1^ with full width at half maximum (FWHM) of 10.8 cm^-1^. Two peaks observed at ~ 950.40 and 973.83 cm^-1^ are also TO peaks. With the lower laser power below 100 mW, only the TO peak at ~ 519.41 cm^-1^ is distinctly observed.


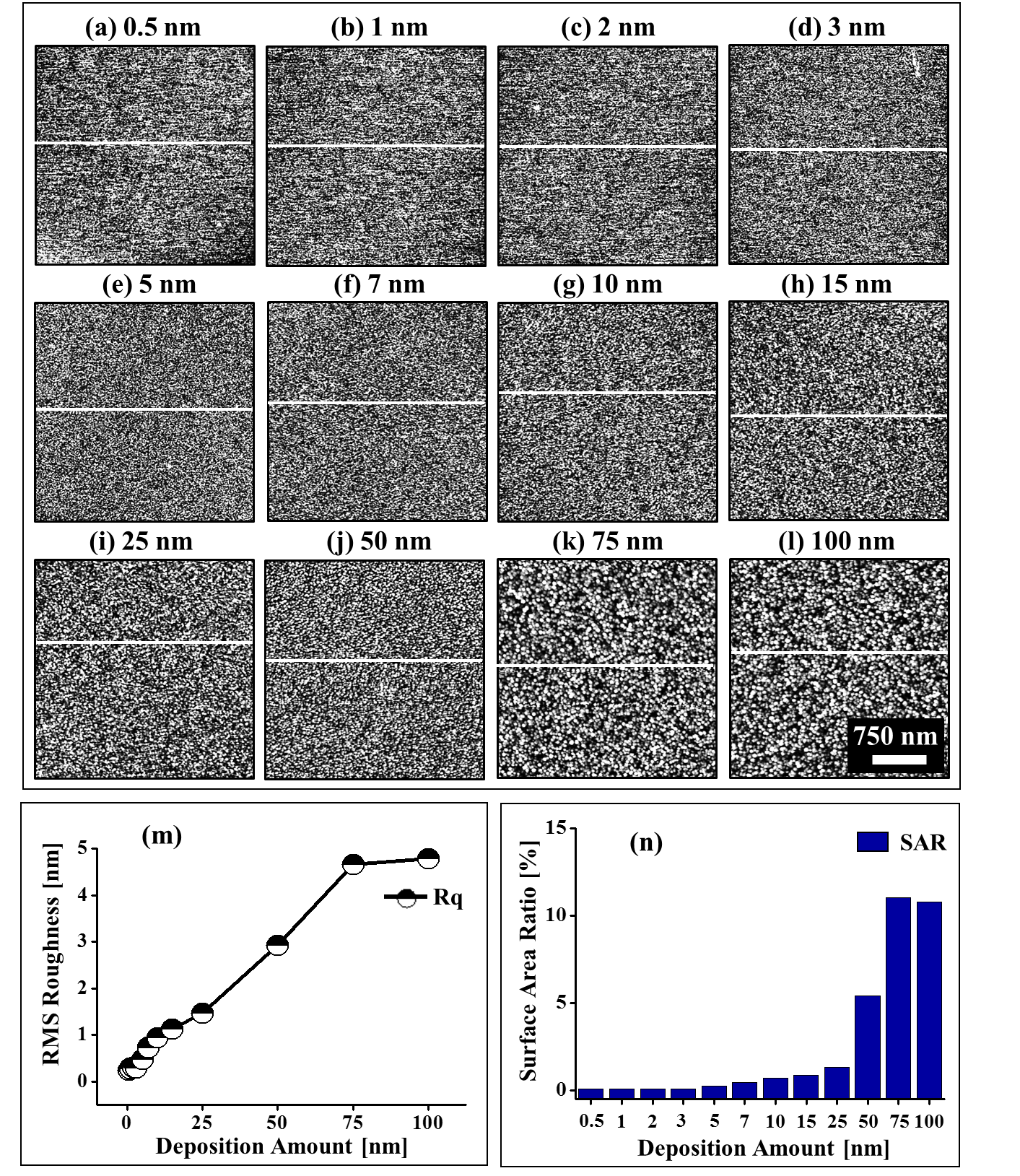


**Figure S2:** Pre-annealed samples with various Pd deposition amount between 0.5 and 100 nm on Si (111). (a) – (l) AFM top-views of 3 × 3 µm^2^. Summary plots of (m) Rq and (n) SAR of pre-annealed samples obtained from the corresponding AFM images (3 × 3 µm^2^) in Fig. S2.


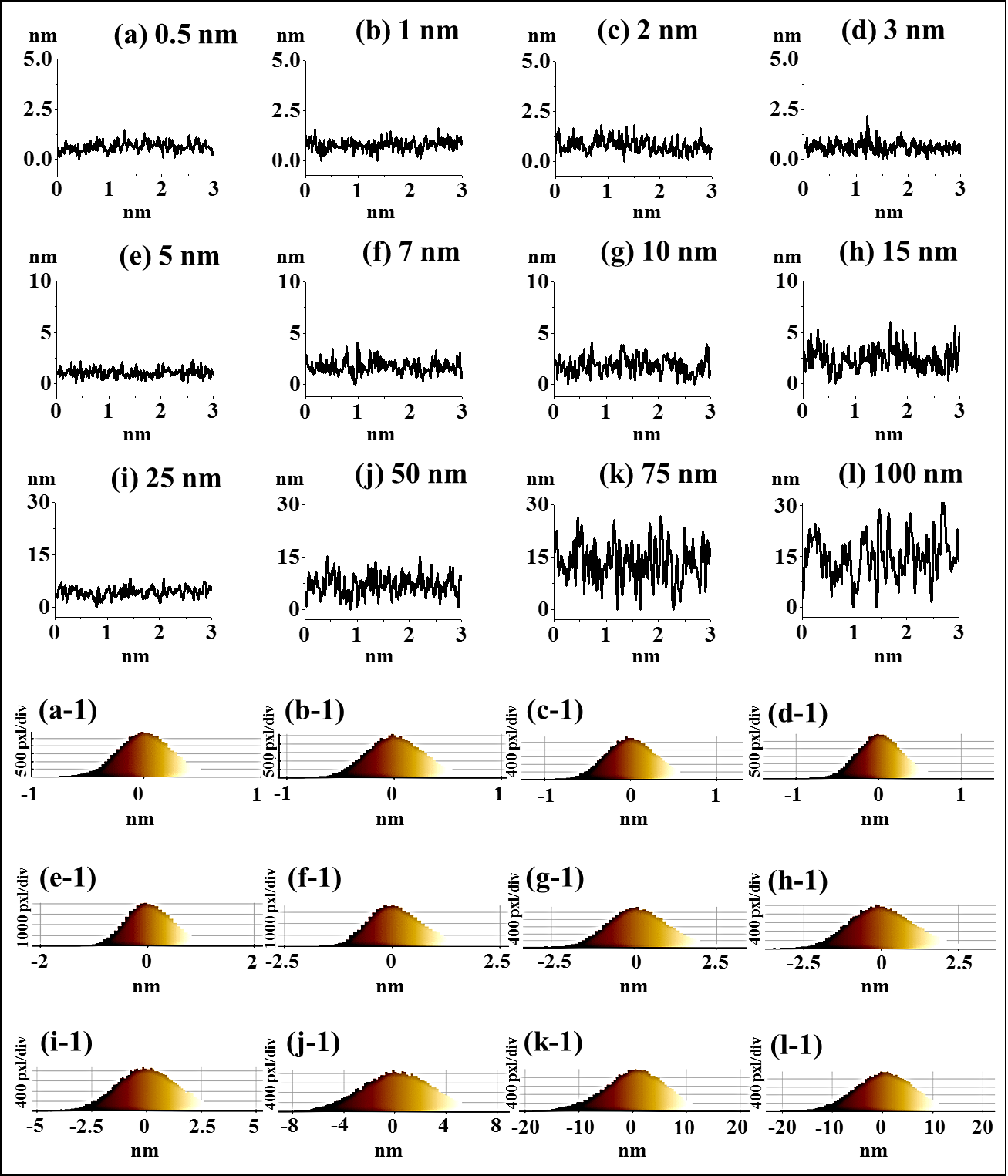


**Figure S3:** (a) – (l) Cross-sectional line-profiles of pre-annealed samples in Fig. S2. (a-1) – (l-1) Corresponding height distribution histogram.


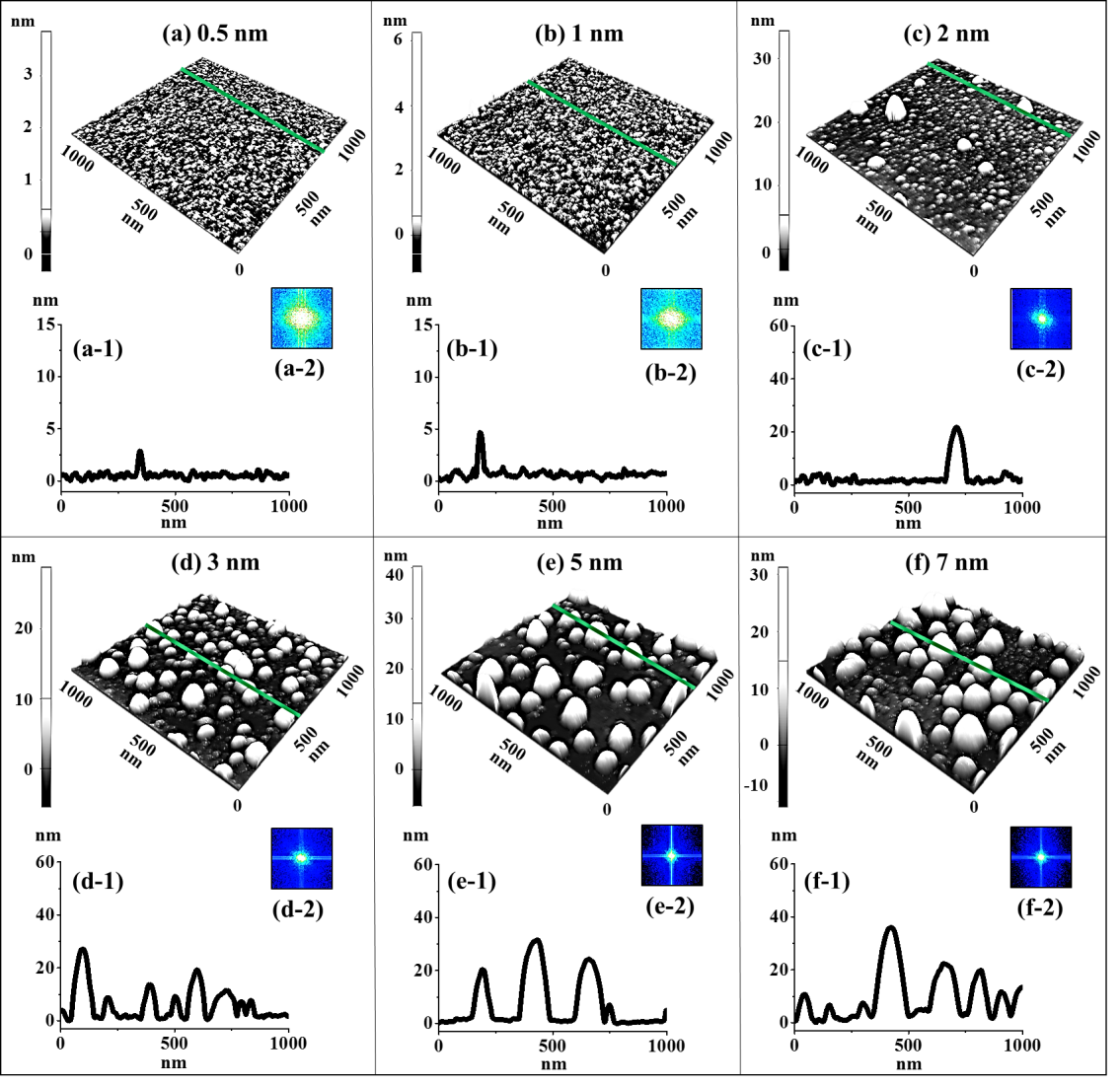


**Figure S4:** Formation of various size, configuration and density of Pd nanostructures on Si (111) with the control of Pd deposition amount between 0.5 and 7 nm. The annealing was performed at 575 ^o^C for 450 s. (a) – (f) AFM side-views of 1 × 1 µm^2^. (a-1) - (f-1) Cross-sectional line-profiles. (a-2) - (f-2) Corresponding 2-D FFT power spectra.


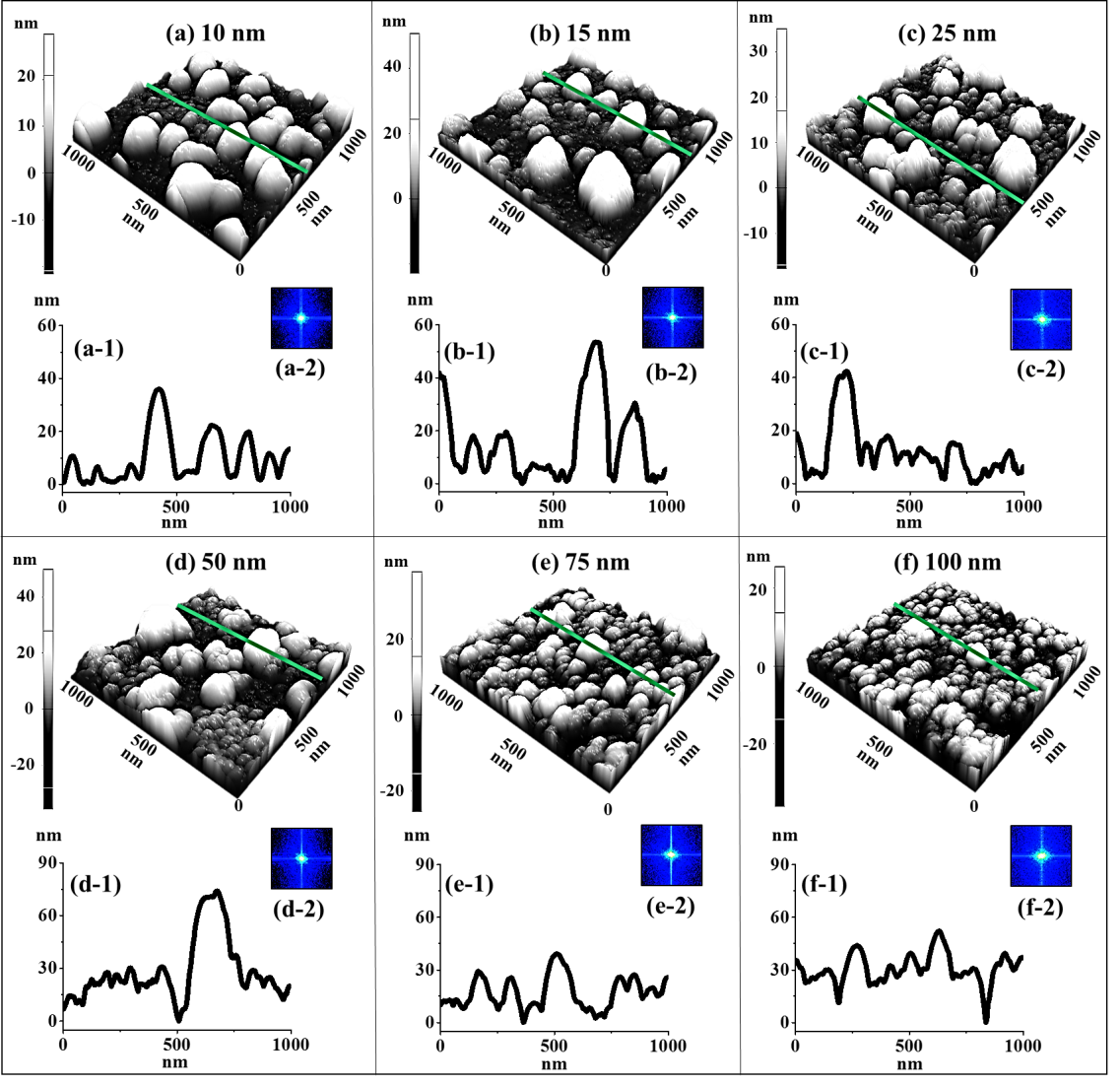


**Figure S5:** Effect of the high deposition amount between 10 and 100 nm on the evolution of Pd nanostructures on Si (111) annealed at 575 ^o^C for 450 s. (a) – (f) AFM side-views of of 1 × 1 µm^2^. (a-1) - (f-1) Cross-sectional line-profiles. (a-2) - (f-2) 2-D FFT power spectra.


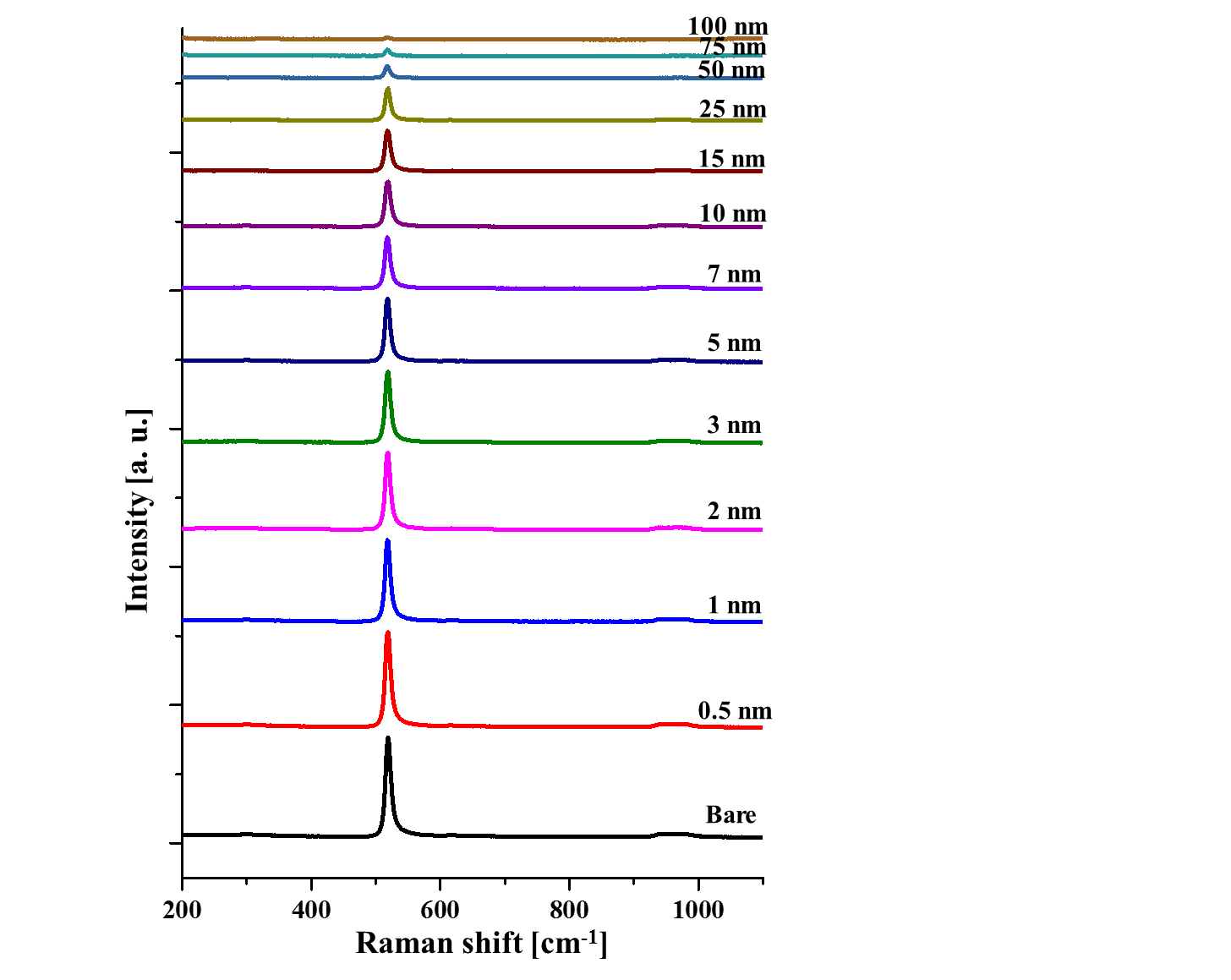


**Figure S6:** Raman spectra of the Pd NPs on Si measured at room temperature using a CW 532 nm laser at 100 mW with deposition amount between 0.5 and 100 nm annealed at 575 ^o^C for 450 s.

**
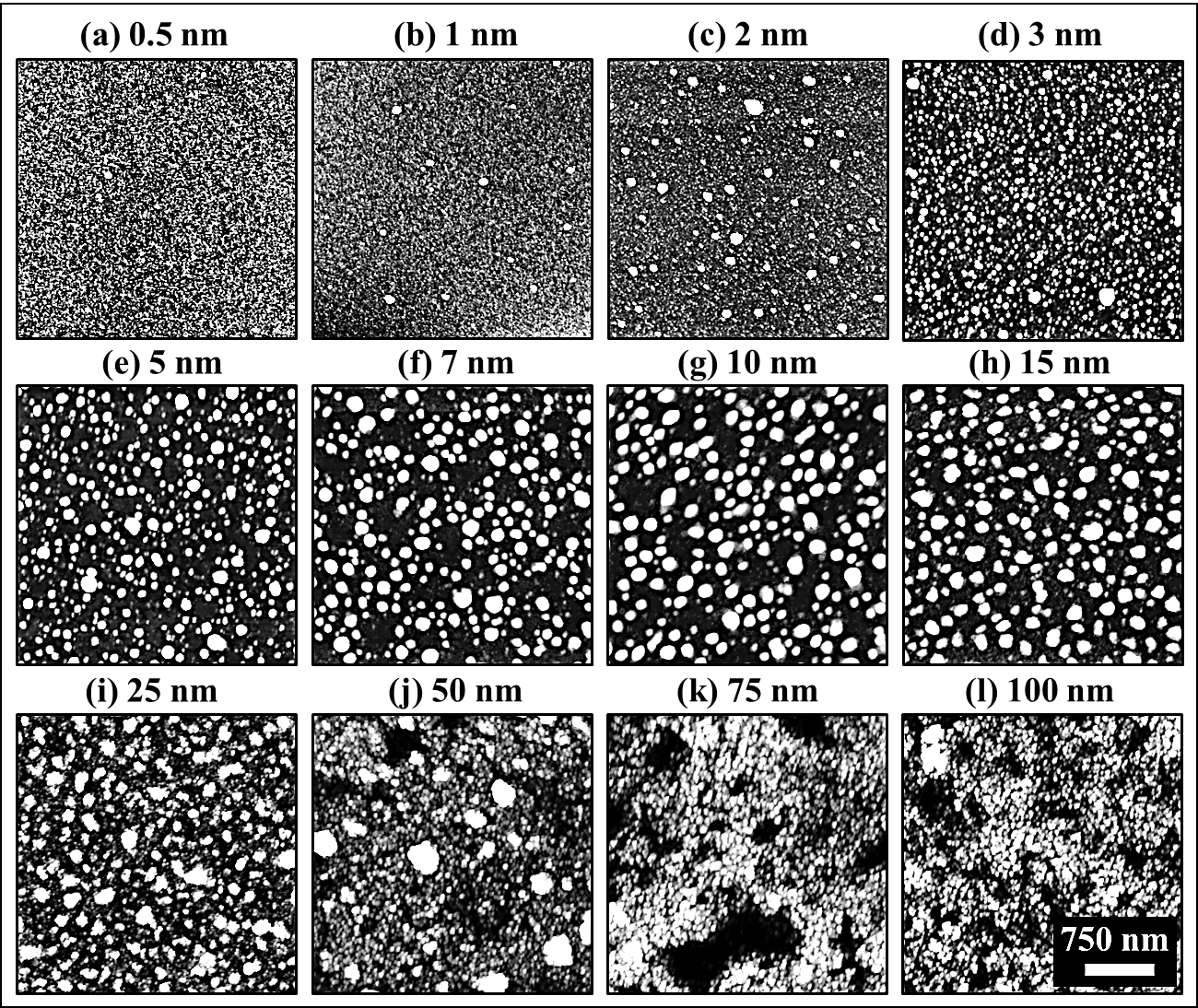
**

**Figure S7:** The effect of comparatively high annealing temperature (700 ^o^C for 450 s) on the evolution of Pd nanostructures on Si (111) with various amount of Pd thickness between 0.5 and 100 nm. (a) – (l) AFM top-views of 3 × 3 µm^2^.

**
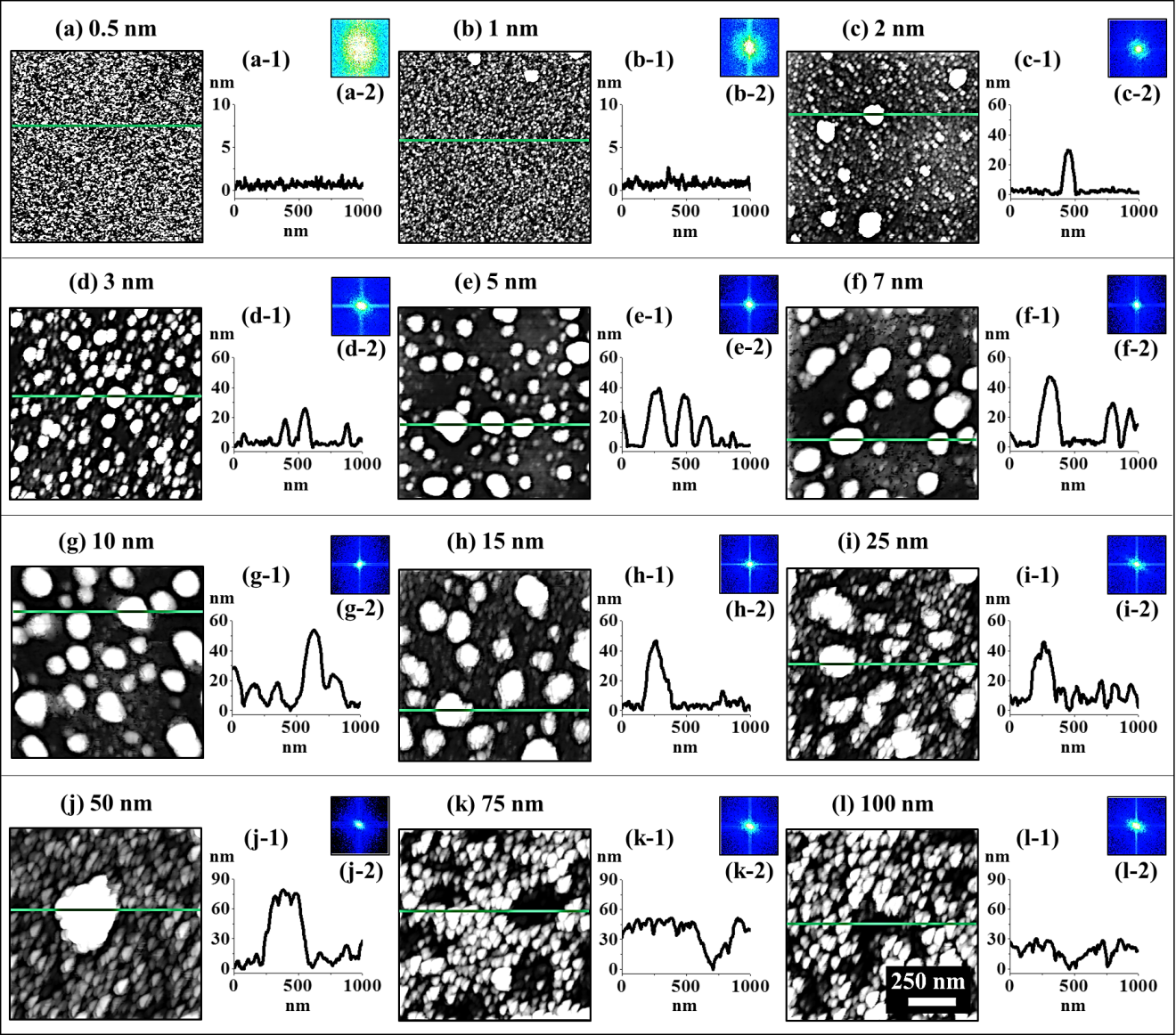
**

**Figure S8:** Evolution of various size, configuration and density of Pd nanostructures on Si (111). The Pd deposition amount was systematically controlled between 0.5 and 100 nm, and followed by the annealing at 700 ^o^C for 450 s. (a) – (l) AFM top-views of 1 × 1 µm^2^. (a-1) - (l-1) Cross-sectional line-profiles. (a-2) - (l-2) 2-D FFT power spectra.

**
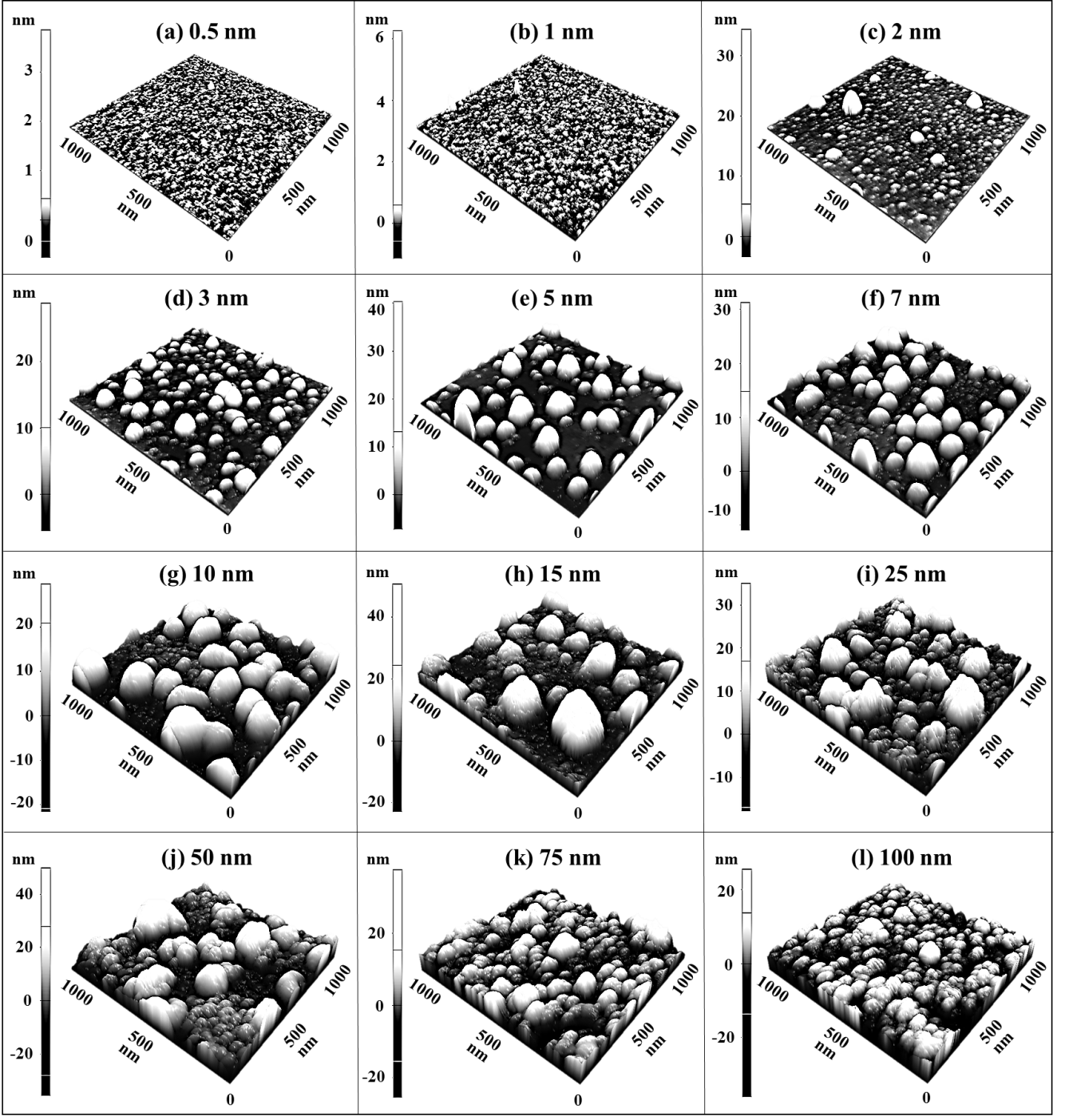
**

**Figure S9:** AFM side-views (1 × 1 µm^2^) of Pd nanostructures after the annealing of various Pd deposition amount between 0.5 and 100 nm on Si (111) at 700 ^o^C for 450 s.

**
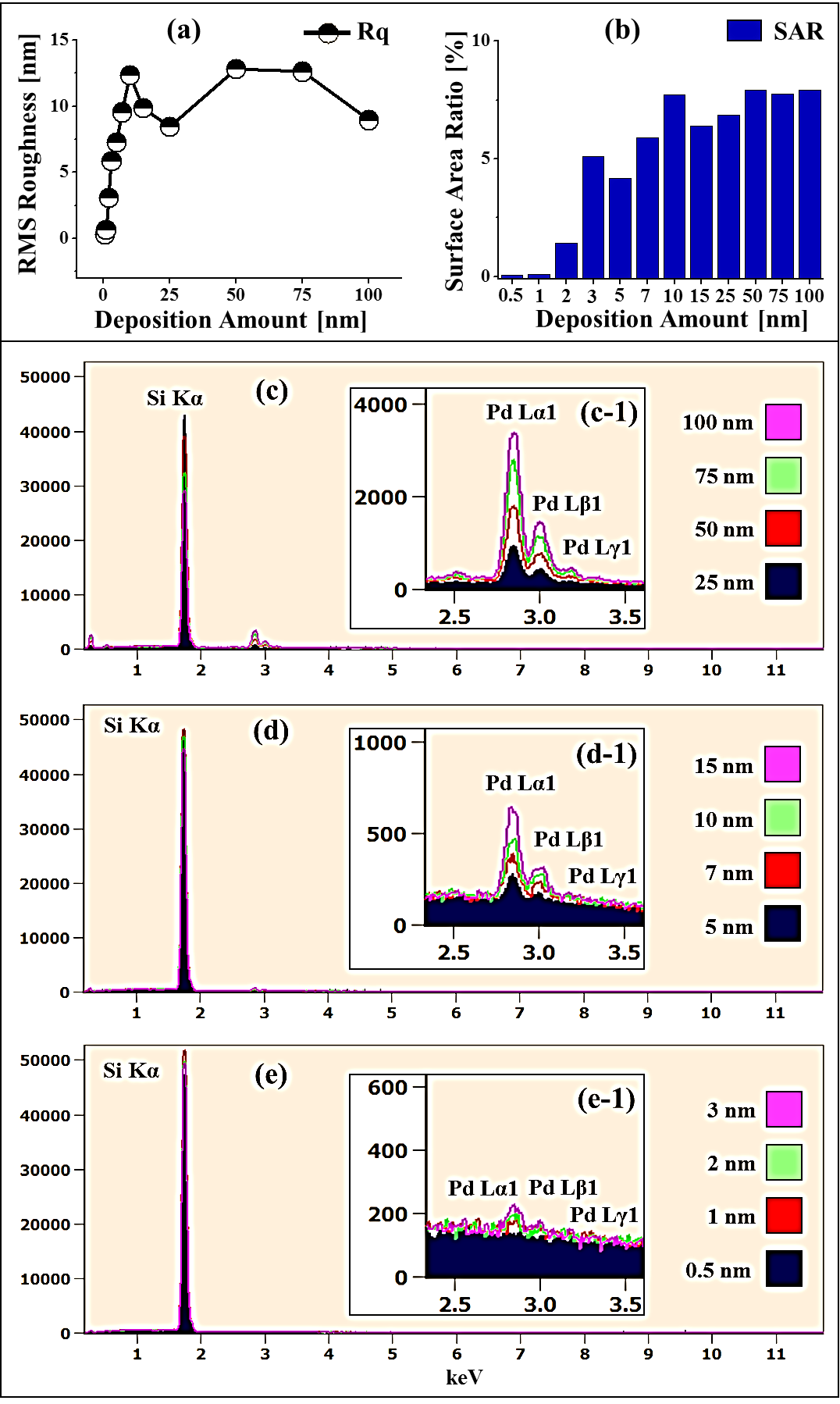
**

**Figure S10:** (a) – (b**)** Plots of Rq and SAR of various sample annealed at 700 ^o^C for 450 s. (c) – (e) EDS spectra showing the gradual increase of counts with respect to the deposition amount. (c-1) – (e-1) Enlarged spectra between 2.3 and 3.6 keV.

**
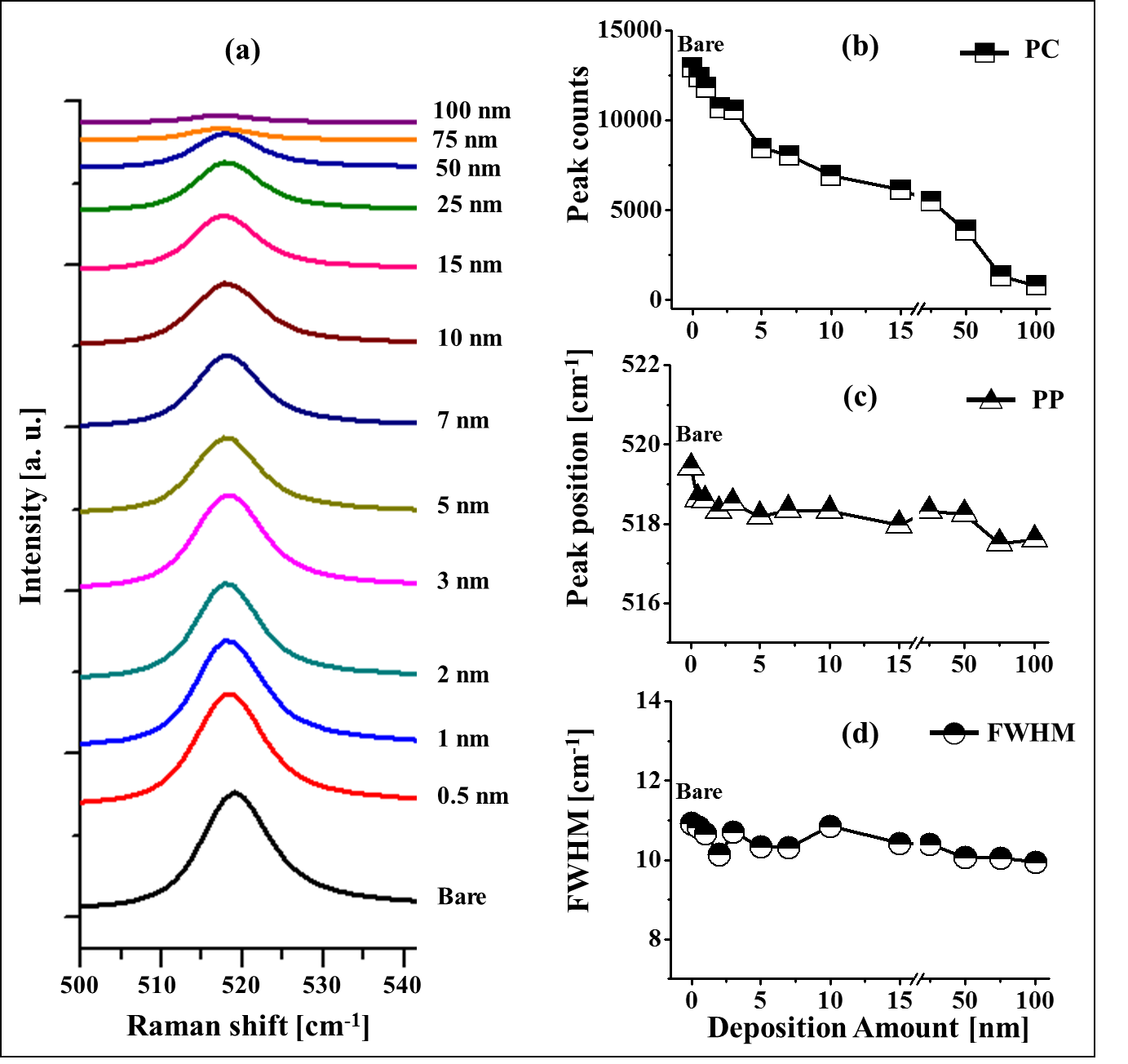
**

**Figure S11:** Raman spectra of various Pd nanostructures fabricated on Si (111) with the systematic variation of deposition amount between 0.5 and 100 nm annealed at 700 ^o^C for 450 s. (a) Raman spectra measured between 500 and 540 cm^-1^. Plots of (b) peak counts (PC), (c) peak position (PP) and (c) Full width at half maximum (FWHM) as function of deposition amount.

**
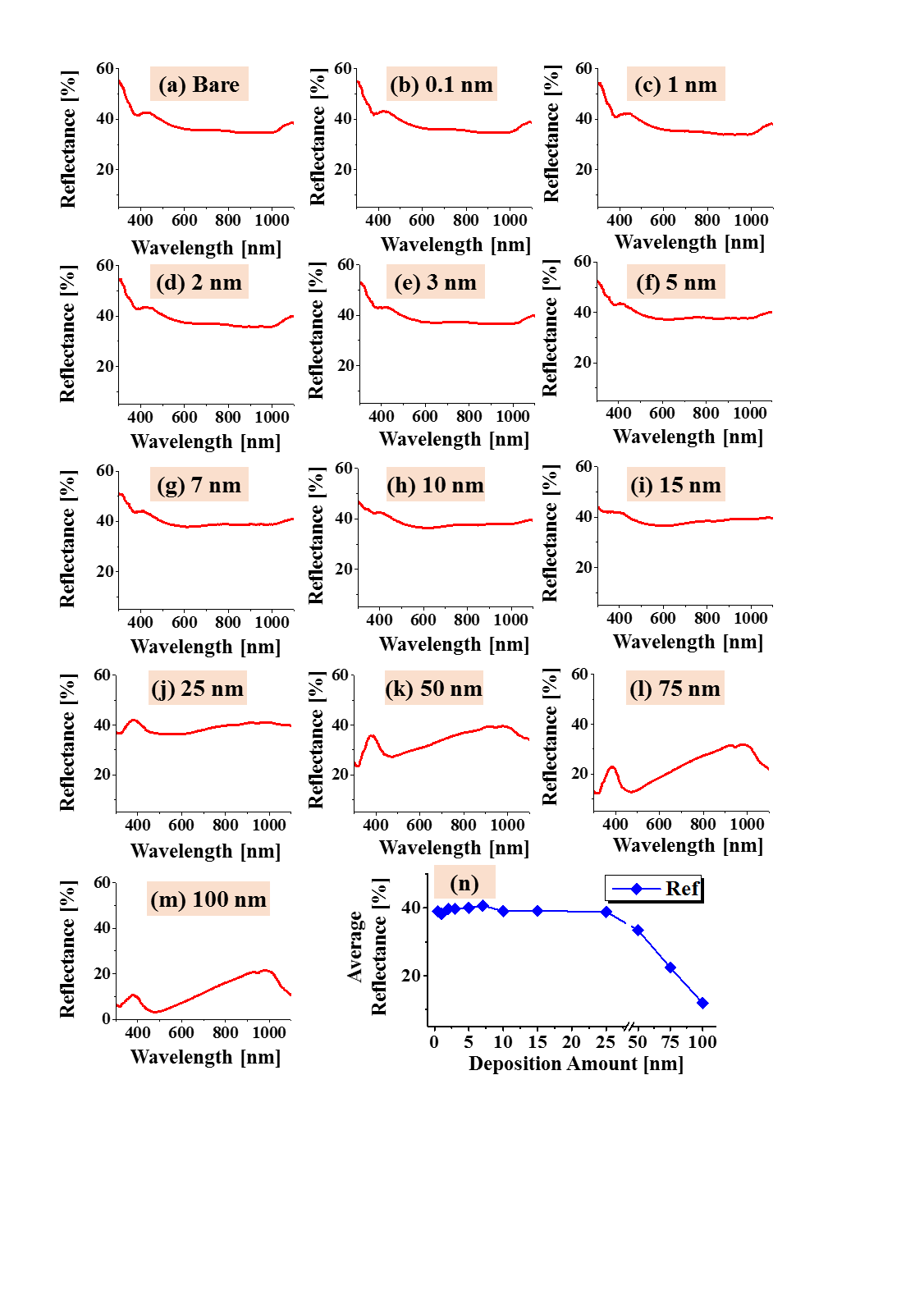
**

**Figure S12:** Reflectance spectra of (a) bare silicon (111) and (b) – (m) samples fabricated with Pd nanostructures based on the deposition amount variation between 0.1 and 100 nm at 700 °C for 450 s. (n) Summary plot of average reflectance.


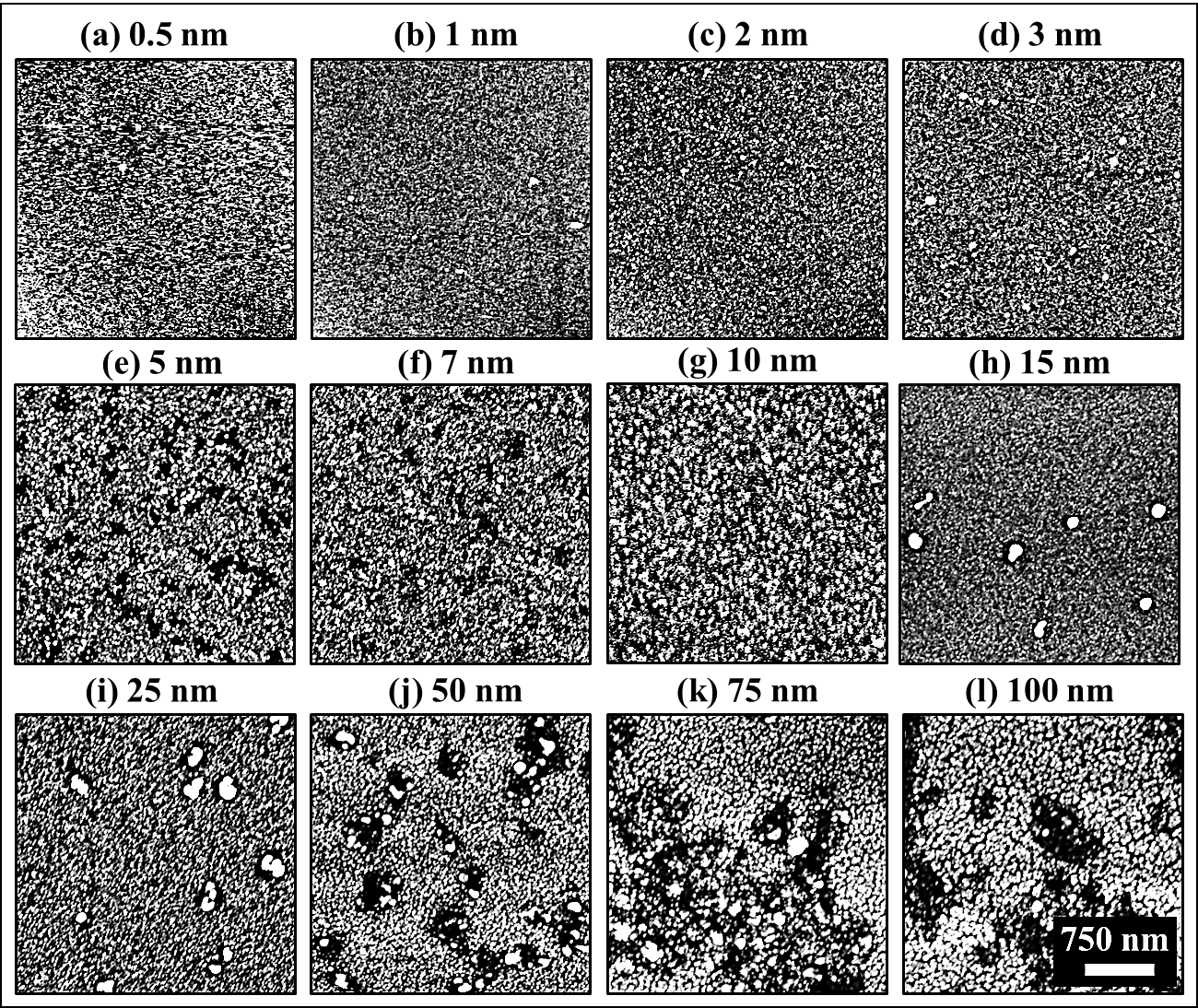


**Figure S13:** Formation of various Pd nanostructures due to the limited diffusion of Pd adatoms at the lower annealing temperature of 450 ^o^C for 450 s with various amount of Pd deposition between 0.5 and 100 nm. (a) – (l) AFM top-views of 3 × 3 µm^2^.


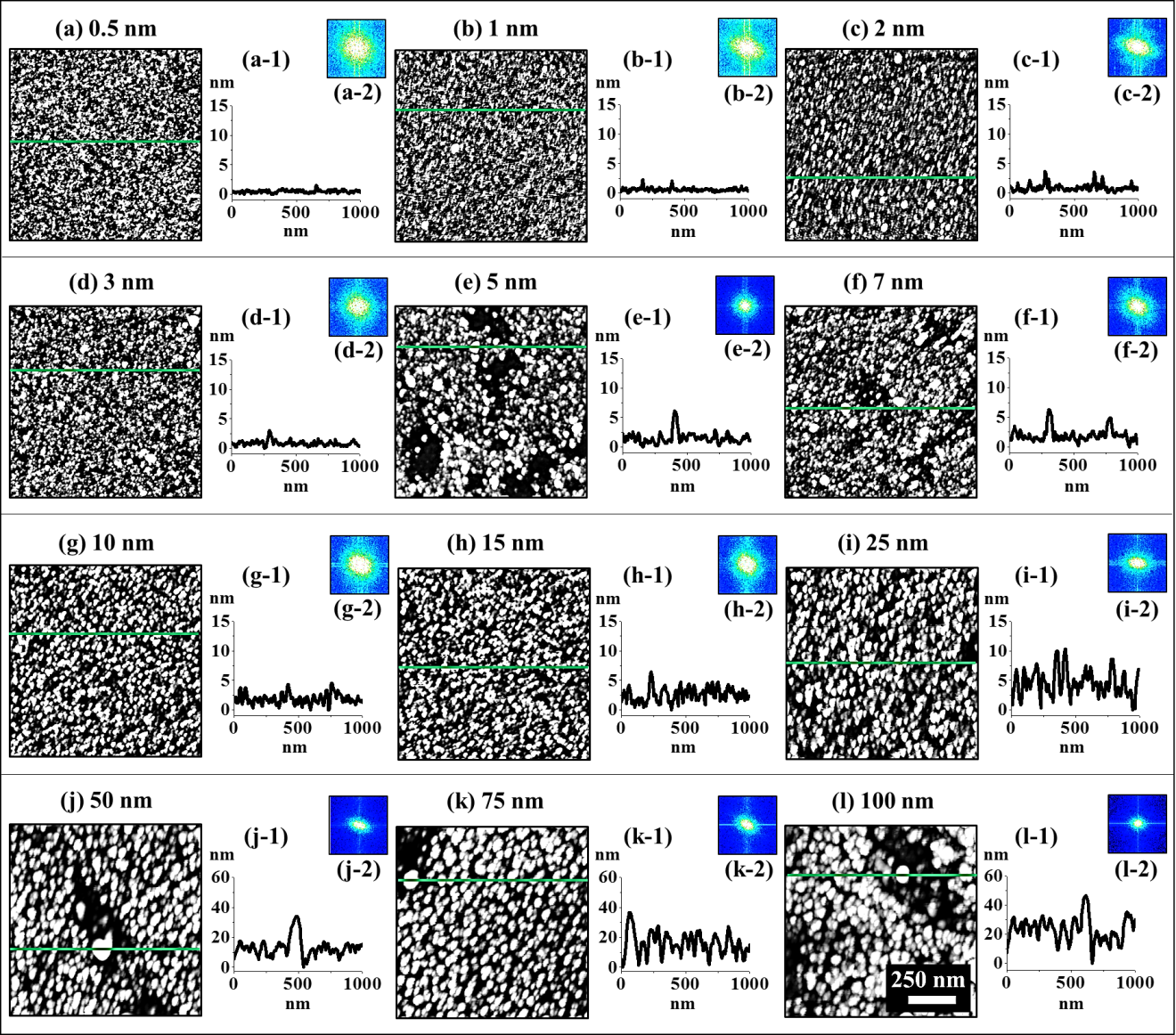


**Figure S14:** (a) – (l) AFM top-views (1 × 1 µm^2^) of various Pd nanostructures on Si (111). The Pd deposition amount was systematically varied between 0.5 and 100 nm, and followed by the annealing at 450 ^o^C for 450 s. (a-1) - (l-1) Cross-sectional line-profiles. (a-2) - (l-2) 2-D FFT power spectra.


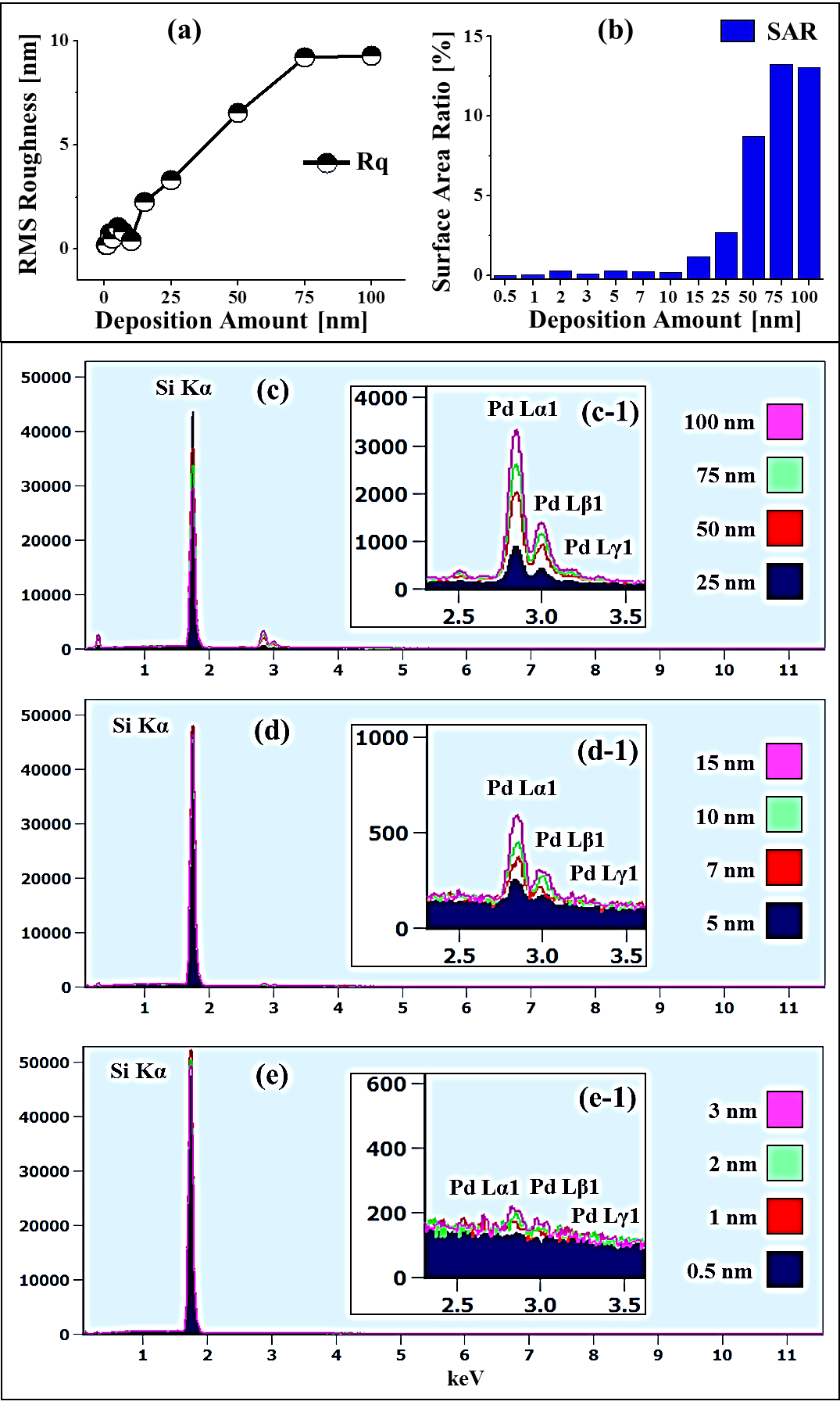


**Figure S15:** (a) – (b**)** Plots of Rq and SAR obtained from the AFM images in Fig. S12 and (c) – (e) EDS spectra. (0.5 and 100 nm at 450 ^o^C for 450 s)


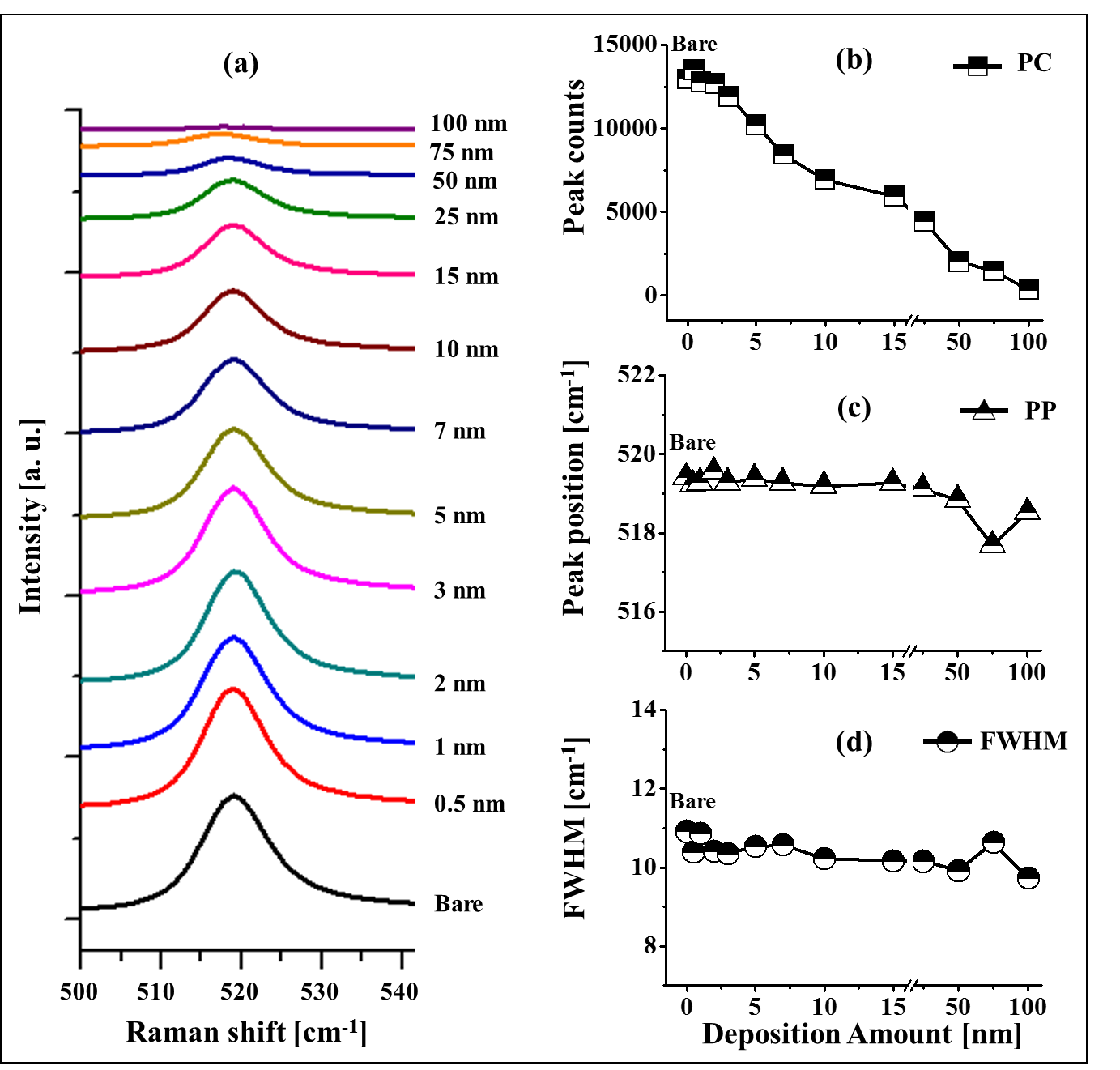


**Figure S16:** Raman spectra of samples with Pd deposition amount between 0.5 and 100 nm on Si (111) and annealing at 450 ^o^C for 450 s. (a) Raman spectra measured between 500 and 540 cm^-1^. Plots of (b) PC, (c) PP and (d) FWHM as function of deposition amount. The value at 0 nm correspond to bare Si (111) in (b) - (d).

**
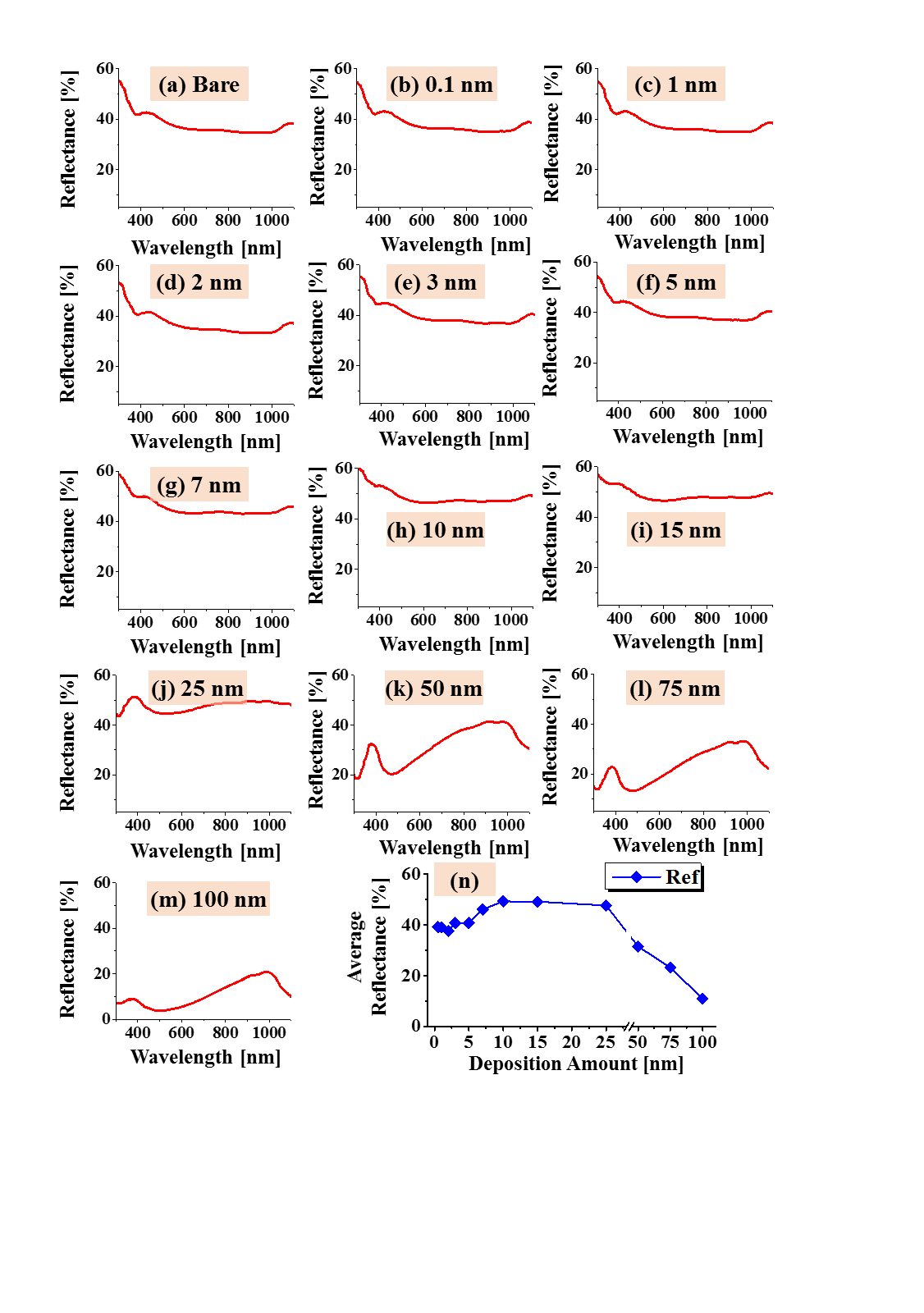
**

**Figure S17:** Reflectance spectra of the (a) bare silicon (111) and (b) – (m) samples with Pd nanostrucutres fabricated by varying deposition amount from 0.5 to 100 nm as shown in Fig. S12. (n) Summary plots of average reflectance.


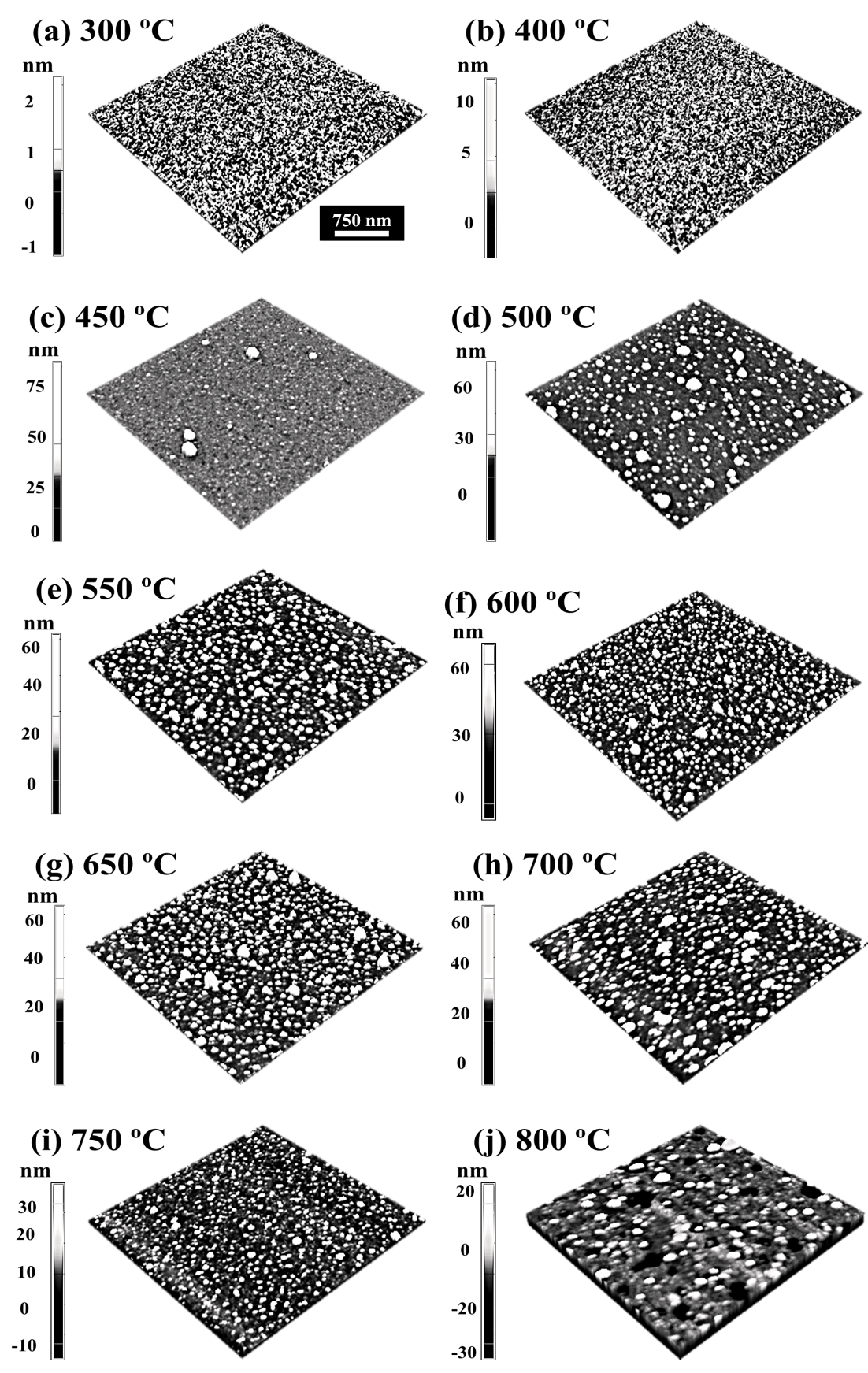


**Figure S18:** Formation of various Pd nanostructures such as: small pits and grains, NPs and holes on Si (111) with the 5 nm of Pd thickness. The fabrication was controlled by the annealing temperature between 300 and 800 °C for 450 s. AFM 3-D side-views (3 × 3 µm^2^) of the samples.

**
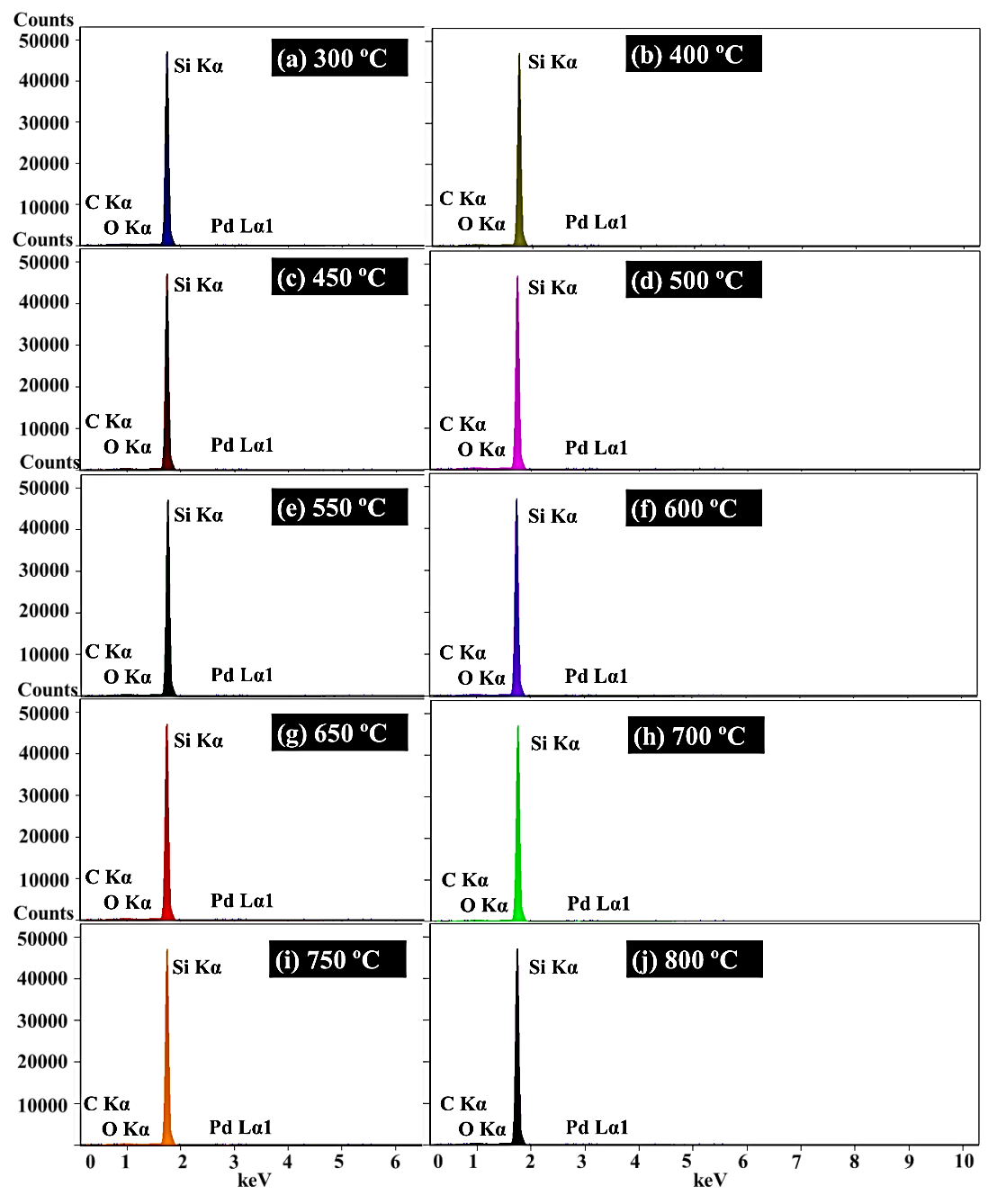
**

**Figure S19:** (a) – (j) Full range EDS spectra of the samples with a variation of the annealing temperature between 300 and 800 °C for 450 s with the deposition amount of 5 nm.

**Table S1:** Summary of RMS roughness (Rq) and surface area ratio (SAR) of samples with Pd deposition amount between 0.5 and 100 nm on Si (111) and after annealing at 575 ^o^C for 450 s. The data were obtained from the 3 × 3 µm^2^ AFM images.

| **Deposition Amount**  **[nm]** | **As deposited** | | **575 ^o^C Annealing** | |
| --- | --- | --- | --- | --- |
|  | **Rq [nm]** | **SAR [%]** | **Rq [nm]** | **SAR [%]** |
| **0.5** | 0.24 | 0.048 | 0.15 | 0.029 |
| **1** | 0.29 | 0.071 | 0.55 | 0.047 |
| **2** | 0.31 | 0.083 | 2.13 | 0.772 |
| **3** | 0.29 | 0.075 | 6.47 | 4.056 |
| **5** | 0.47 | 0.209 | 8.86 | 4.983 |
| **7** | 0.73 | 0.442 | 8.89 | 5.326 |
| **10** | 0.95 | 0.684 | 10.62 | 6.317 |
| **15** | 1.13 | 0.849 | 11.66 | 7.187 |
| **25** | 1.47 | 1.294 | 10.05 | 8.863 |
| **50** | 2.93 | 5.413 | 11.78 | 8.289 |
| **75** | 4.67 | 11.007 | 9.04 | 7.801 |
| **100** | 4.79 | 10.766 | 7.98 | 6.707 |

**Table S2:** Summary of Raman peak counts (PC), peak position (PP) and full width at half maximum (FWHM) of corresponding samples with deposition amount variation from 0.5 to 100 nm on Si (111) annealed at 575 ^o^C for 450 s.

| **Deposition Amount  [nm]** | **Peak Counts** | **Peak Position [ cm^-1^]** | **FWHM  [cm^-1^]** |
| --- | --- | --- | --- |
| **Bare** | 12942.39 | 519.4049 | 10.91429 |
| **0.5** | 12596.79 | 518.9388 | 10.81231 |
| **1** | 10721.89 | 518.585 | 10.50681 |
| **2** | 10143.07 | 518.7198 | 10.50884 |
| **3** | 9428.344 | 518.7957 | 10.72691 |
| **5** | 8305.246 | 518.4458 | 10.14705 |
| **7** | 6767.835 | 518.3507 | 10.62587 |
| **10** | 6056.419 | 518.796 | 10.94667 |
| **15** | 5445.43 | 518.6446 | 10.83987 |
| **25** | 4251.998 | 518.7269 | 10.09891 |
| **50** | 1552.461 | 518.1359 | 10.1817 |
| **75** | 803.5939 | 518.3851 | 10.05138 |
| **100** | 261.9897 | 518.0455 | 10.25946 |

**Table S3:** Summay of average height (AH), average width (AW) and average density (AD) of the Pd NPs fabricated between 3 and 15 nm at at 575 ^o^C for 450 s.

| **Deposition**  **Amount [nm]** | **AH**  **[nm]** | **AW**  **[nm]** | **AD**  **[× 10^8^/ cm^2^]** |
| --- | --- | --- | --- |
| **3** | 16.09 | 86.86 | 2.17 |
| **5** | 21.19 | 112.49 | 1.58 |
| **7** | 25.56 | 131.16 | 1.36 |
| **10** | 30.89 | 165.33 | 0.96 |
| **15** | 38.16 | 176.66 | 0.81 |

**Table S4:** Summary of Rq and SAR obtained from the AFM images of various morphology of Pd nanostructures. The Pd deposition amount was systematically varied between 0.5 and 100 nm on Si (111). The annealing was perforemd at 450 and 700 ^o^C for 450 s.

| **Deposition Amount [nm]** | **450 ^o^C Annealing** | | **700 ^o^C Annealing** | |
| --- | --- | --- | --- | --- |
|  | **Rq [nm]** | **SAR [%]** | **Rq [nm]** | **SAR [%]** |
| **0.5** | 0.19 | 0.024 | 0.30 | 0.048 |
| **1** | 0.19 | 0.070 | 0.65 | 0.091 |
| **2** | 0.74 | 0.308 | 3.08 | 1.420 |
| **3** | 0.50 | 0.099 | 5.83 | 5.091 |
| **5** | 1.04 | 0.324 | 7.26 | 4.181 |
| **7** | 0.83 | 0.255 | 9.54 | 5.907 |
| **10** | 0.39 | 0.210 | 12.34 | 7.715 |
| **15** | 2.25 | 1.181 | 9.86 | 6.381 |
| **25** | 3.31 | 2.714 | 8.46 | 6.860 |
| **50** | 6.53 | 8.740 | 12.83 | 7.919 |
| **75** | 9.19 | 13.231 | 12.62 | 7.742 |
| **100** | 9.28 | 13.084 | 8.96 | 7.932 |

**Table S5:** Raman measurement of various morphology of Pd nanostructures with various deposition amount between 0.5 and 100 nm. The peak counts (PC), peak position (PP) and full width at half maximum (FWHM) were changed accordingly with the surface morphology.

| **Deposition Amount [nm]** | **450 ^o^C Annealing** | | | **700 ^o^C Annealing** | | |
| --- | --- | --- | --- | --- | --- | --- |
|  | **PC** | **PP**  **[cm^-1^]** | **FWHM [cm^-1^]** | **PC** | **PP**  **[cm^-1^]** | **FWHM**  **[cm^-1^]** |
| **Bare** | 12942.39 | 519.4049 | 10.91429 | 12942.39 | 519.4049 | 10.91429 |
| **0.5** | 13501.28 | 519.2193 | 10.38699 | 12397.86 | 518.6124 | 10.82784 |
| **1** | 12783.13 | 519.2782 | 10.85253 | 11835.03 | 518.5834 | 10.65454 |
| **2** | 12686.53 | 519.5469 | 10.4092 | 10684.8 | 518.3118 | 10.13237 |
| **3** | 11900.68 | 519.2757 | 10.35947 | 10560.59 | 518.522 | 10.69938 |
| **5** | 10196.48 | 519.3692 | 10.52605 | 8442.953 | 518.1816 | 10.3386 |
| **7** | 8425.922 | 519.2731 | 10.57273 | 8030.054 | 518.3408 | 10.31731 |
| **10** | 6920.393 | 519.1873 | 10.2222 | 6914.507 | 518.3245 | 10.84675 |
| **15** | 5942.842 | 519.2608 | 10.17104 | 6134.001 | 517.9626 | 10.41526 |
| **25** | 4435.627 | 519.1216 | 10.15266 | 5487.487 | 518.3155 | 10.39576 |
| **50** | 2019.162 | 518.8359 | 9.92535 | 3867.4 | 518.23 | 10.05702 |
| **75** | 1452.343 | 517.6769 | 10.62852 | 1319.469 | 517.4951 | 10.04879 |
| **100** | 320.4893 | 518.5194 | 9.73939 | 797.8776 | 517.5922 | 9.9417 |

**Table S6:** Summary of the SAR and Rq of the samples annealed at temperature between 300 and 800 ˚C for 450 s with the deposition amount of 5 nm.

| **Annealing Temperature  [^o^C]** | **SAR [%]** | **Rq [nm]** |
| --- | --- | --- |
| **Bare** | 0.025 | 0.21 |
| **300** | 0.096 | 0.48 |
| **400** | 1.46 | 6.67 |
| **450** | 5.36 | 8.59 |
| **500** | 7.47 | 7.51 |
| **550** | 9.48 | 7.45 |
| **600** | 8.35 | 7.95 |
| **650** | 4.38 | 6.85 |
| **700** | 1.81 | 2.96 |
| **750** | 1.41 | 2.65 |
| **800** | 0.025 | 0.21 |

**Table S7:** Summary of AH, AW and AD of the Pd NPs fabricated between 500 and 700 ºC for 450 s with constant deposition amount of 5 nm.

| **Annealing Temperature [ºC]** | **AH**  **[nm]** | **AW**  **[nm]** | **AD**  **[× 10^8^/ cm^2^]** |
| --- | --- | --- | --- |
| **500** | 18.49 | 79.64 | 34.22 |
| **550** | 2028 | 83.75 | 39.22 |
| **600** | 18.95 | 71.53 | 43 |
| **650** | 25.81 | 90.36 | 26.55 |
| **700** | 21.75 | 102.68 | 27.22 |

**Table S8:** Summary of peak counts (PC), peak position (PP) and full width at half maximum (FWHM) of the Raman spectrum of the samples annealed at various annealing temperature between 300 and 800 ˚C with 5 nm deposition amount and 450 s annealing duration.

| **Annealing Temperature**  **[^o^C]** | **PC** | **PP [cm^-1^]** | **FWHM [cm^-1^]** |
| --- | --- | --- | --- |
| **Bare** | 11305.6 | 519.71 | 10.15 |
| **300** | 8903.9 | 519.26 | 8.9 |
| **400** | 8954.5 | 518.82 | 9.38 |
| **450** | 9173.3 | 519.75 | 9.51 |
| **500** | 10175.6 | 519.26 | 9.15 |
| **550** | 9953.13 | 519.2 | 9.63 |
| **600** | 9614.7 | 518.38 | 9.22 |
| **650** | 9621.4 | 519.7 | 9.18 |
| **700** | 9668.2 | 519.65 | 9.14 |
| **750** | 10535.1 | 518.59 | 9.49 |
| **800** | 10819.1 | 519.15 | 8.85 |

**Table S9:** Summary of average reflectance of the Pd nanostructures on Si with various Pd deposition amount from 0.5 to 100 nm annealed at distinct temperatures: 450, 575 and 700 °C for 450 s.

| **Deposition**  **Amounts [nm]** | **Average Reflectance [%]** | | |
| --- | --- | --- | --- |
|  | **450 °C** | **575 °C** | **700 °C** |
| **Bare** | 38.81 | 39.04 | 38.63 |
| **0.5** | 39.15 | 38.96 | 38.93 |
| **1** | 39.06 | 38.50 | 38.24 |
| **2** | 37.60 | 40.10 | 39.69 |
| **3** | 40.72 | 40.62 | 39.71 |
| **5** | 40.64 | 40.49 | 40.02 |
| **7** | 46.02 | 40.69 | 40.65 |
| **10** | 49.23 | 44.32 | 39.07 |
| **15** | 49.11 | 40.72 | 39.10 |
| **25** | 47.58 | 28.56 | 38.85 |
| **50** | 31.50 | 35.45 | 33.39 |
| **75** | 23.12 | 26.27 | 22.37 |
| **100** | 10.90 | 15.71 | 11.92 |

**Table S10:** Average reflectance of the samples with the constant 5 nm Pd thickness annealed for 450 s at varying temperature from 300 to 800 °C.

| **Annealing Temperature  [ºC]** | **Average Reflectance  [%]** |
| --- | --- |
| **300** | 41.6 |
| **400** | 42.1 |
| **450** | 42.12 |
| **500** | 40.13 |
| **550** | 40.31 |
| **600** | 41.4 |
| **650** | 41.17 |
| **700** | 40.82 |
| **750** | 39.38 |
| **800** | 31.1 |
